# Fractionation of human spermatogenic cells using STA-PUT gravity sedimentation and their miRNA profiling

**DOI:** 10.1038/srep08084

**Published:** 2015-01-30

**Authors:** Yun Liu, Minghui Niu, Chencheng Yao, Yanan Hai, Qingqing Yuan, Yang Liu, Ying Guo, Zheng Li, Zuping He

**Affiliations:** 1 State Key Laboratory of Oncogenes and Related Genes, Renji- Med X Clinical Stem Cell Research Center, Ren Ji Hospital, School of Medicine, Shanghai Jiao Tong University, Shanghai, China; 2 Department of Urology, Ren Ji Hospital, School of Medicine, Shanghai Jiao Tong University, Shanghai Human Sperm Bank, Shanghai Institute of Andrology, 145 Shangdong Road, Shanghai 200001, China; 3 Shanghai Key Laboratory of Assisted Reproduction and Reproductive Genetics, Shanghai 200127, China; 4 Shanghai Key Laboratory of Reproductive Medicine, Shanghai 200025, China

## Abstract

Human spermatogenic cells have not yet been isolated, and notably, their global miRNA profiles remain unknown. Here we have effectively isolated human spermatogonia, pachytene spermatocytes and round spermatids using STA-PUT velocity sedimentation. RT-PCR, immunocytochemistry and meiosis spread assays revealed that the purities of isolated human spermatogonia, pachytene spermatocytes, and round spermatids were 90%, and the viability of these isolated cells was over 98%. MiRNA microarrays showed distinct global miRNA profiles among human spermatogonia, pachytene spermatocytes, and round spermatids. Thirty-two miRNAs were significantly up-regulated whereas 78 miRNAs were down-regulated between human spermatogonia and pachytene spermatocytes, suggesting that these miRNAs are involved in the meiosis and mitosis, respectively. In total, 144 miRNAs were significantly up-regulated while 29 miRNAs were down-regulated between pachytene spermatocytes and round spermatids, reflecting potential roles of these miRNAs in mediating spermiogenesis. A number of novel binding targets of miRNAs were further identified using various softwares and verified by real-time PCR. Our ability of isolating human spermatogonia, pachytene spermatocytes and round spermatids and unveiling their distinct global miRNA signatures and novel targets could provide novel small RNA regulatory mechanisms mediating three phases of human spermatogenesis and offer new targets for the treatment of male infertility.

Spermatogenesis is a process by which male germline stem cells self-renew and differentiate to male gametes, namely, spermatozoa that transmit genetic information to subsequent generations. In general, spermatogenesis comprises three main phases, including the mitosis of spermatogonia, meiosis of spermatocytes, and spermiogenesis by which round spermatids change their shapes to become enlongated spermatids. To isolate spermatogonia, pachytene spermatocytes, and round spermatids with high purities and viabilities from human testis tissues is essential for elucidating molecular mechanisms controlling mitosis, meiosis and spermiogenesis. Male germ cells can be separated from testis tissues by several approaches, such as the velocity sedimentation, magnetic-activated cell sorting (MACS), and fluorescence-activated cell sorting (FACS). The latter two approaches depend on biochemical markers of male germ cells, and cell purity and viability are largely affected by the specificity of the chosen antibodies. We have isolated male germline stem cells in rodents and humans using MACS^1^. Currently, male germ cells, including spermatogonia, pachytene spermatocytes and round spermatids, have been separated by STA-PUT velocity sedimentation in mice^2^,^3^. However, the separation of spermatogonia, pachytene spermatocytes and round spermatids has not yet been achieved and identified in humans.

Spermatogenesis is precisely regulated by genetic and epigenetic factors. Although much progress has been made on uncovering the mechanisms underlying spermatogenesis in rodents, very little is known about epigenetic and genetic regulation of spermatogonia, spermatocytes and spermatids in human, due to the difficulties in obtaining human testis tissues. Recently microRNAs (miRNAs) have been identified as a novel class of short single-stranded small RNA molecules (~18–22 nucleotides). MiRNAs regulate gene expression through binding and targeting mRNAs for degradation or suppressing translation^4^, and notably, miRNAs may control 30% of all genes in human genomes^5^. A number of studies reflect that miRNAs have essential functions in various kinds of biological processes, including cellular proliferation^6^, differentiation^7^,^8^ and apoptosis^9^. Differential miRNA expression profiling was identified in mouse male germ cells, including spermatogonia, pachytene spermatocytes and round spermatids^3^. It has been reported that several miRNAs in the miRNA 17–92 cluster are abundantly expressed in mouse gonocytes^10^, and miRNA-21 has been shown to mediate the self-renewal of male germline stem cells^11^. We have recently demonstrated that miRNA-20 and miRNA-106a are required for the proliferation of mouse male germline stem cells^12^. These studies illustrate that miRNAs play critical roles in regulating rodent spermatogenesis. Nevertheless, global miRNA profiles in human male germ cells and roles of miRNAs in mediating human spermatogenesis remain to be defined.

There are distinct cell types of spermatogonia and different biochemical phenotypes between humans and rodents. In human and other primates, spermatogonia are classified as the A_dark_, A_pale_ and type B cells^13^,^14^,^15^, whereas mouse spermatogonia are grouped as the A_s_, A_pr_, A_al_, A_1_–A_4_, intermediate and type B cells. Notably, human spermatogonia share some but not all phenotypes with rodent spermatogonia^1^. As an example, POU5F1 (also known as Oct-4) is expressed specifically in mouse spermatogonia^16^,^17^; however, human spermatogonia are negative for POU5F1^1^. Since cell types and biochemical phenotypes of human male germ cells are distinct from rodents, it is of unusual significance to separate human spermatogenic cells and to uncover the signatures and targets of miRNAs controlling different phases of human spermatogenesis. In this study, we have for the first time isolated human spermatogonia, pachytene spermatocytes and round spermatids from testis tissues with high purities and viabilities using STA-PUT velocity sedimentation. We uncovered distinct global miRNA profiles among human spermatogonia, pachytene spermatocytes and round spermatids using miRNA microarrays and identified their binding targets. Siginficantly, this study could provide novel RNA regulatory mechanisms in regulating human spermatogenesis and offer new targets for treating male infertility.

## Results

### Isolation and morphological characteristics of human spermatogonia, pachytene spermatocytes and round spermatids

**Figure 1:**
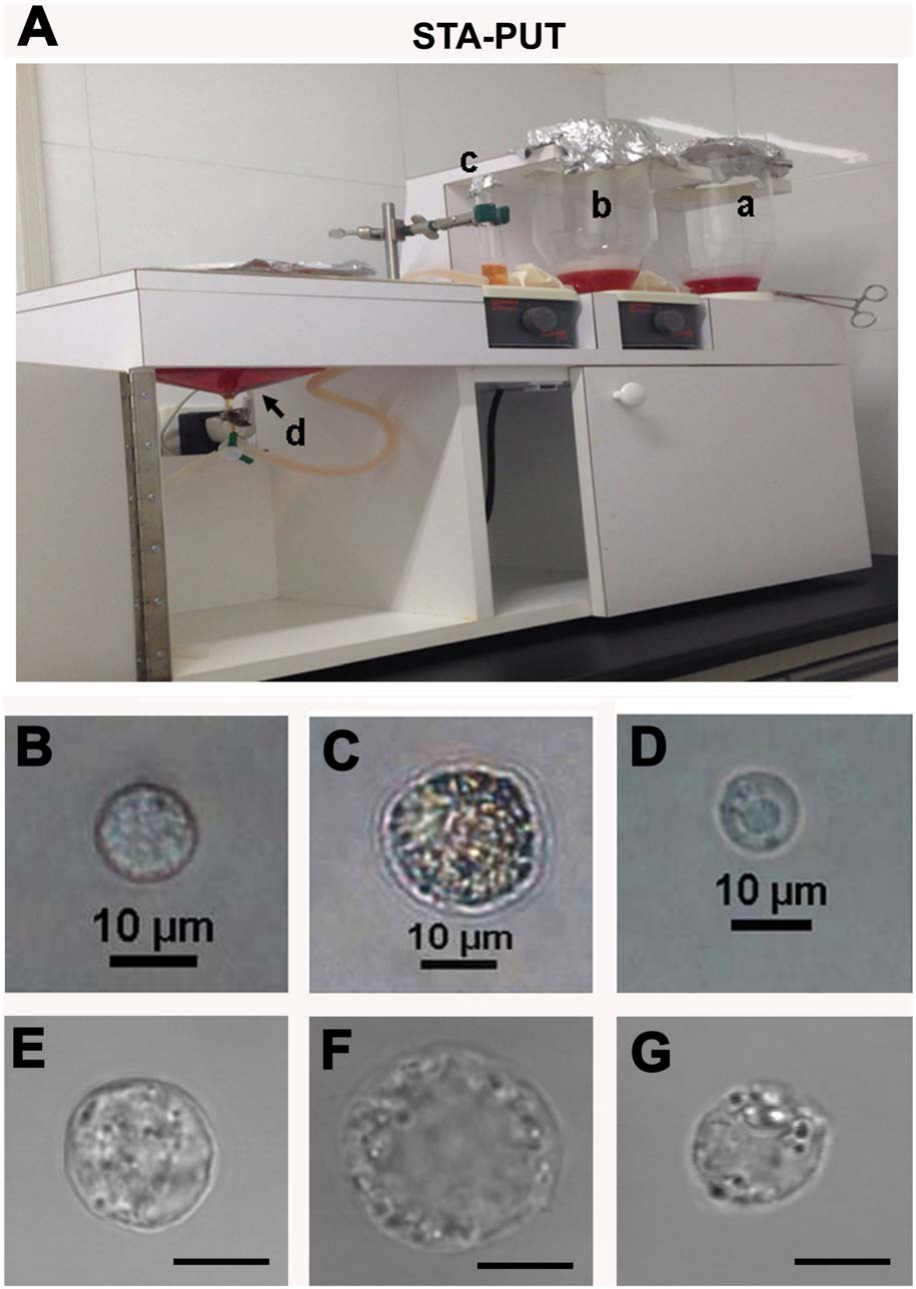
Isolation and morphological characteristics of human spermatogonia, pachytene spermatocytes and round spermatids. (A), STA-PUT apparatus was utilized to separate human spermatogonia, pachytene spermatocytes and round spermatids using 4%, 2% and 0.5% BSA. Notes: a, gradient glass chamber with 4% BSA; b, gradient glass chamber with 2% BSA; c, cell loading chamber with 0.5% BSA; d, standard sedimentation chamber. (B–D), Phase-contrast microscope showed the morphological characteristics of the freshly isolated human spermatogonia (B), pachytene spermatocytes (C) and round spermatids (D). (E–G), DIC microscope revealed the appearance of the freshly isolated human spermatogonia (E), pachytene spermatocytes (F) and round spermatids (G). Scale bars in E, F and G = 10 μm. The data shown in (B–G) were representatives from eight independent experiments of thirty patients.

We first separated human spermatogonia, pachytene spermatocytes and round spermatids from human testicular tissues of obstructive azoospermia (OA) patients with normal spermatogenesis (Supplementary Fig. 1A and 1B) using STA-PUT apparatus (Fig. 1A) via the velocity sedimentation. The isolated cells were identified and characterized based upon their morphological and phenotypic characteristics. Human spermatogonia were collected consecutively from cell fractions 10 to 15 in 15 ml of the centrifuge tubes. Under a phase-contrast microscope and a differential interference contrast (DIC) microscope, these cells were spherical in shape and had large round or ovoid nuclei and a diameter ranging from 9 to 12 μm (Fig. 1B and 1E). Pachytene spermatocytes were obtained by pooling cell fractions 3 to 7 in 15 ml of the centrifuge tubes. With respects to morphological features, human pachytene spermatocytes ranged from 14 to 16 μm in diameter, and they possessed patchy condensed chromatin (Fig. 1C and 1F). Meanwhile, human round spermatids were obtained by collecting cell fractions 18–30 in 15 ml of the centrifuge tubes. Under a phase-contrast microscope and a DIC microscope, human round spermatids were uniform and smaller cells with round nuclei, and they had a diameter ranging from 6 to 8 μm (Fig. 1D and 1G). The viabilities of the freshly isolated human spermatogonia, pachytene spermatocytes and round spermatids were over 98%, as evidenced by trypan blue exclusion of these cells (data not shown).

### Phenotypic characteristics of the freshly isolated human spermatogonia

**Figure 2:**
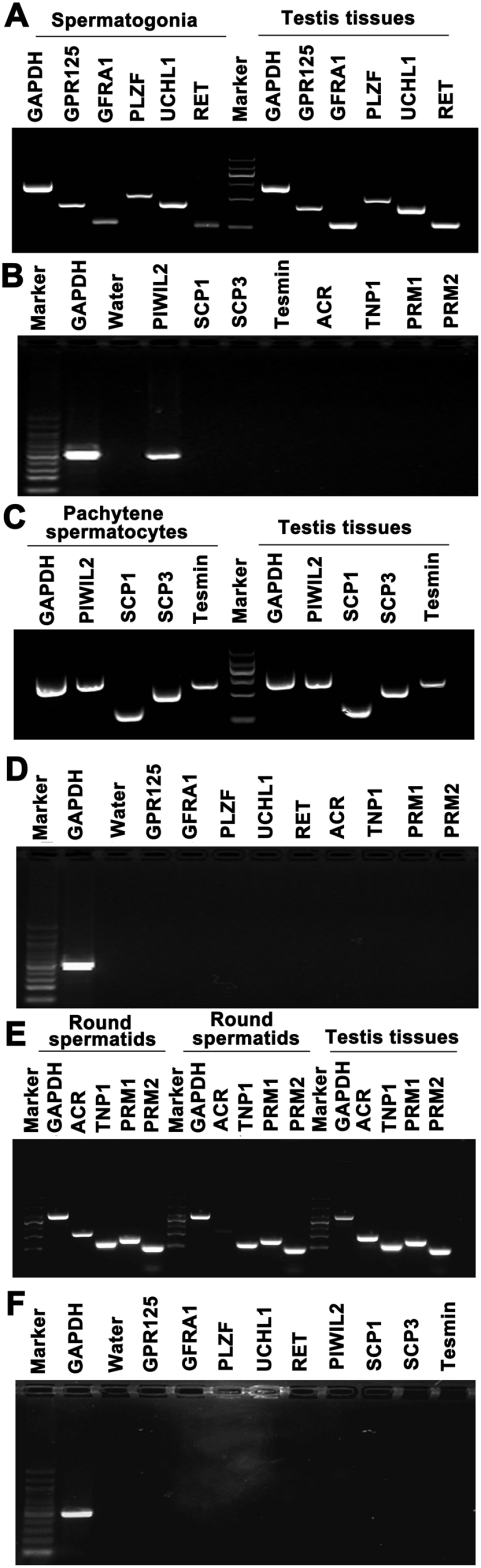
The gene expression of the isolated human spermatogonia, pachytene spermatocytes and round spermatids. (A–B), RT-PCR revealed the transcripts of GPR125, GFRA1, PLZF, UCHL1, RET, SCP1, SCP3, Tesmin, PIWIL2, ACR, TNP1, PRM1 and PRM2 in the freshly isolated human spermatogonia. (C–D), RT-PCR showed the mRNA of SCP1, SCP3, Tesmin, PIWIL2, GPR125, GFRA1, PLZF, UCHL1, RET, ACR, TNP1, PRM1 and PRM2 in the freshly isolated human pachytene spermatocytes. (E–F), RT-PCR displayed the transcripts of ACR, TNP1, PRM1, PRM2, GPR125, GFRA1, PLZF, UCHL1, RET, PIWIL2, SCP1, SCP3 and Tesmin in human round spermatids isolated from human testis tissues. The expression of these genes in human testis tissues served as positive controls. GAPDH served as a loading control of total RNA, and water without DNA was employed as a negative control. The data shown in (A–F) were representatives from eight independent experiments of thirty patients.

**Figure 3:**
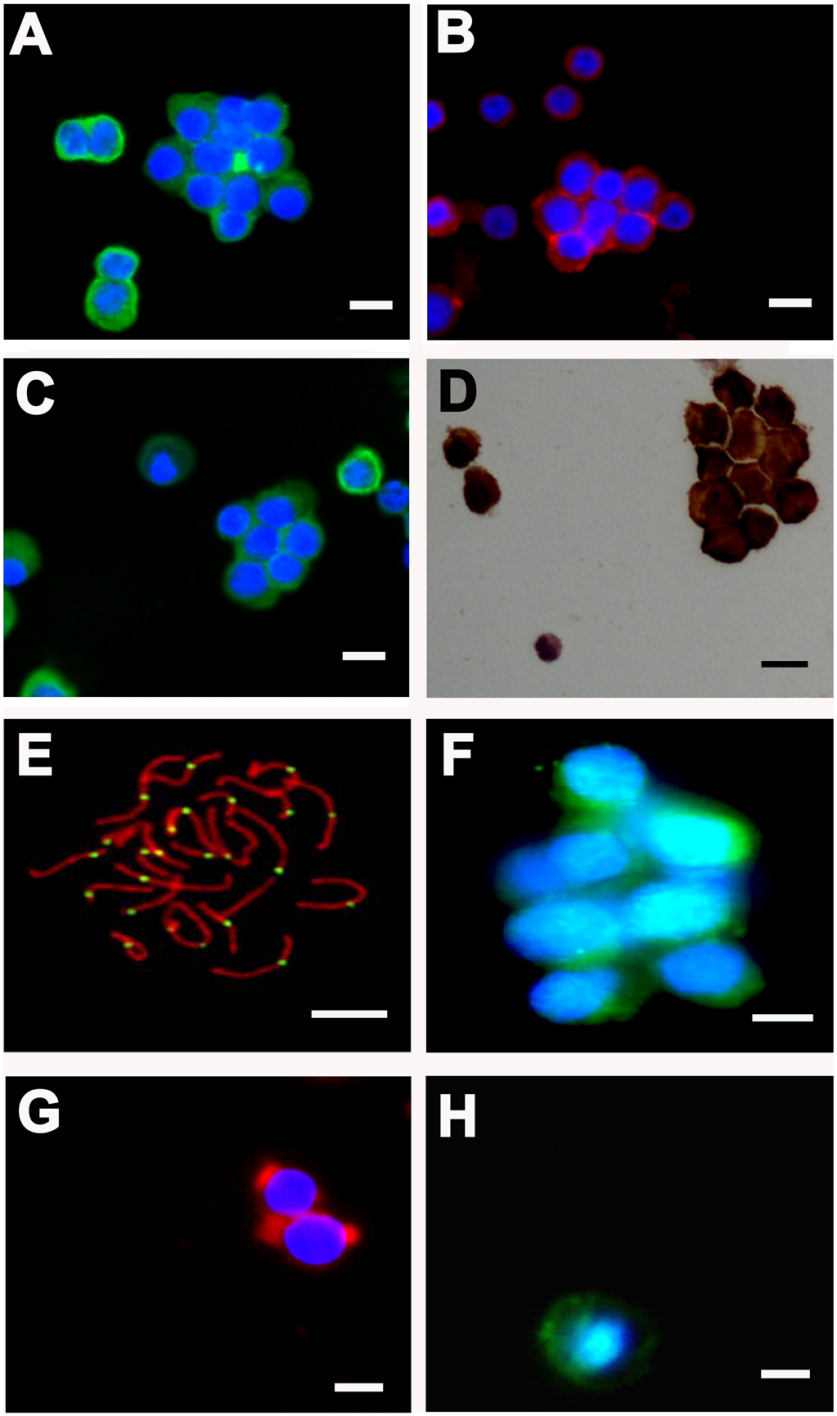
The protein expression of the isolated human spermatogonia, pachytene spermatocytes and round spermatids. (A–D), Immunocytochemistry revealed the expression of GPR125 (A), GFRA1 (B), UCHL1 (C) and MAGEA4 (D) in the freshly isolated human spermatogonia. Scale bars in A–D = 10 μm. (E), Meiosis spread assays displayed the expression of SCP3 (red fluorescence) and CREST (green fluorescence) in the freshly isolated human pachytene spermatocytes. Scale bar in E = 5 μm. (F), Immunocytochemistry showed the expression of SCP3 in the freshly isolated human pachytene spermatocytes. Scale bar in F = 10 μm. (G–H), Immunocytochemistry revealed the expression of Acrosin (G) and Protamine 2 (H) in the isolated human round spermatids. Scale bars in G and H = 5 μm. The data shown in (A–H) were representatives from eight independent experiments of thirty patients.

We next examined the phenotypic features of the freshly isolated human spermatogonia, pachytene spermatocytes and round spermatids by the STA-PUT method. To assess the purities of freshly isolated cells, a number of biochemical hallmarks for human spermatogonia, pachytene spermatocytes and round spermatids were employed in this study. We have recently found that GPR125, GFRA1, MAGEA4, PLZF, and UCHL1 are expressed in human spermatogonia including SSCs^1^. We first characterized the identity of the freshly isolated cells using various markers for human spermatogonia. RT-PCR analyses showed that GPR125, GFRA1, PLZF, UCHL1 and RET transcripts were present in the cells obtained from the fractions 10 to 15 (Fig. 2A), whereas SCP1, SCP3, Tesmin, ACR, TNP1, PRM1 and PRM2 were undetected in them (Fig. 2B). Immunocytochemical analysis revealed that 90% of the cells from the fractions 10 to 15 were positive for GPR125 (Fig. 3A), GFRA1 (Fig. 3B), UCHL1 (Fig. 3C) and MAGEA4 (Fig. 3D). Collectively, these results suggest that the freshly isolated cells are phenotypically human spermatogonia.

### Biochemical features of the freshly isolated human pachytene spermatocytes

Meiotic recombination is essential for the segregation of homologous chromosome and the formation of normal haploid gametes. Meiosis spread assay has been recently developed and widely used to probe meiotic recombination on spread preparations of spermatocytes in humans or animals^18^,^19^. SCP3 is a major components of the lateral elements of synaptonemal complexes (SCs) and a sister chromatid arm cohesin during mammalian meiosis I, which has been used to visualize SCs. The kinetochore can be detected with CREST antisera to visualize centromeric regions. Tesmin is regarded as a nuclear and cytoplasm protein that is specifically expressed in spermatocytes^20^,^21^,^22^, while SCP1 is selectively expressed at the meiotic prophase of spermatocytes^23^,^24^. RT*-*PCR analysis displayed that the transcripts of SCP1, SCP3 and Tesmin were present in the cells derived from fractions 3 to 7 (Fig. 2C); in contrast, GPR125, GFRA1, PLZF, UCHL1, RET, ACR, TNP1, PRM1 and PRM2 were not detected in these cells (Fig. 2D). Both SCP3 and CREST have been regarded as markers for pachytene spermatocytes. Meiosis spread assays reflected that the freshly isolated cells from fractions 3 to 7 were coexpressed SCP3 and CREST (Fig. 3E). Immunocytochemistry further displayed that 92% of these cell fraction were positive for SCP3 (Fig. 3F). Considered together, these data implicate that these freshly isolated cells are human pachytene spermatocytes in phenotype.

### Phenotypic characteristics of the freshly human round spermatids

We also examined the phenotype of the freshly isolated cells from fractions 18–30 using numerous markers for spermatids. During spermiogenesis, Acrosin is a major protease present in the acrosome of mature mammalian spermatozoa, while TNP1 (transition protein 1) and Protamine 2 are spermatid-specific proteins^25^. RT-PCR assays revealed that the expression of ACR (acrosin), TNP1, PRM1 (protamine 1) and PRM2 (protamine 2) was present in the cells from fractions 18 to 30 (Fig. 2E), whereas the transcripts of GPR125, GFRA1, PLZF, UCHL1, RET, PIWIL2, SCP1, SCP3 and Tesmin were hardly detected in these cell fractions (Fig. 2F). Immunofluorescence revealed that 95% of these cells were positive for Acrosin (Fig. 3G) and Protamine 2 (Fig. 3H). Taken together, our results indicate that these freshly isolated cells are human round spermatids in biochemical characteristics.

### Global miRNA profiles in human spermatogonia, pachytene spermatocytes and round spermatids

**Figure 4:**
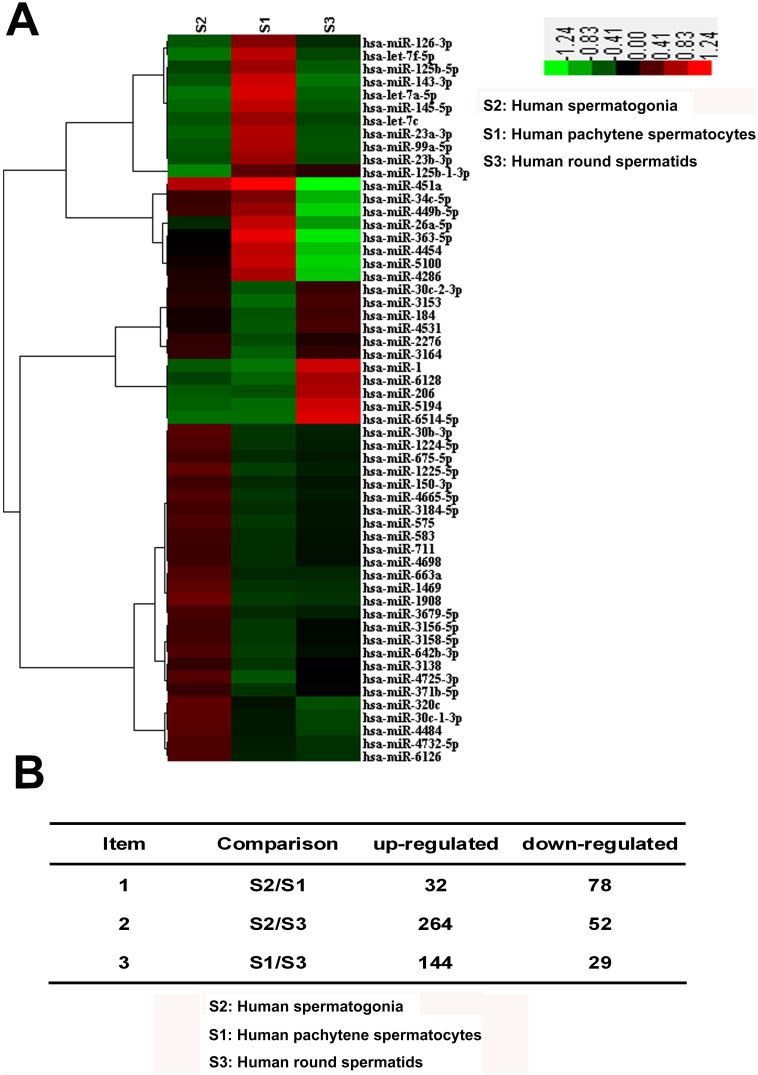
Distinct miRNA expression profiles among human spermatogonia, pachytene spermatocytes and round spermatids. (A), Hierarchical clustering showed the differentially expressed miRNAs among human spermatogonia, pachytene spermatocytes and round spermatids. A total of 599 differentially expressed miRNAs was found from 2,400 microRNA microarray database, based on 1.5-fold and greater difference among human spermatogonia, pachytene spermatocytes and round spermatids. Up-regulated and down-regulated genes were represented in red and green colors, respectively. (B), The number of differentially expressed miRNAs was shown among human spermatogonia, pachytene spermatocytes and round spermatids. The data shown in (A–B) were representatives from two independent experiments of twenty patients.

After isolation and characterization of human spermatogonia, pachytene spermatocytes and round spermatids, total RNA was extracted from these cells and its quality was checked by gel imaging (Supplementary Fig. 2A) and electropherogram assays (Supplementary Fig. 2B). MiRNA microarrays were performed to compare global miRNA profiles among human spermatogonia, pachytene spermatocytes and round spermatids. Based on 2,400 miRNAs in the miRNA microarray database, there were 1,806 miRNAs present in human spermatogonia, pachytene spermatocytes and round spermatids. Hierarchical clustering analysis revealed distinct miRNA expression profiles among human spermatogonia, pachytene spermatocytes and round spermatids (Fig. 4A), and there were totally 599 differentially expressed miRNAs with 1.5-fold changes or more among these cells. Specifically, 32 miRNAs were significantly up-regulated whereas 78 miRNAs were down-regulated between human spermatogonia and pachytene spermatocytes (Fig. 4B, Supplementary Tables 1 and 2), suggesting that these miRNAs may regulate the meiosis and mitosis, respectively. In addition, 144 miRNAs were significantly up-regulated (Fig. 4B, Supplementary Table 3), whereas 29 miRNAs were down-regulated between pachytene spermatocytes and round spermatids (Fig. 4B, Supplementary Table 4), reflecting the potential roles of these miRNAs in controlling spermiogenesis. Furthermore, there were totally 316 miRNAs that were differentially expressed between human spermatogonia and round spermatids (Fig. 4B, Supplementary Tables 5 and 6).

**Figure 5:**
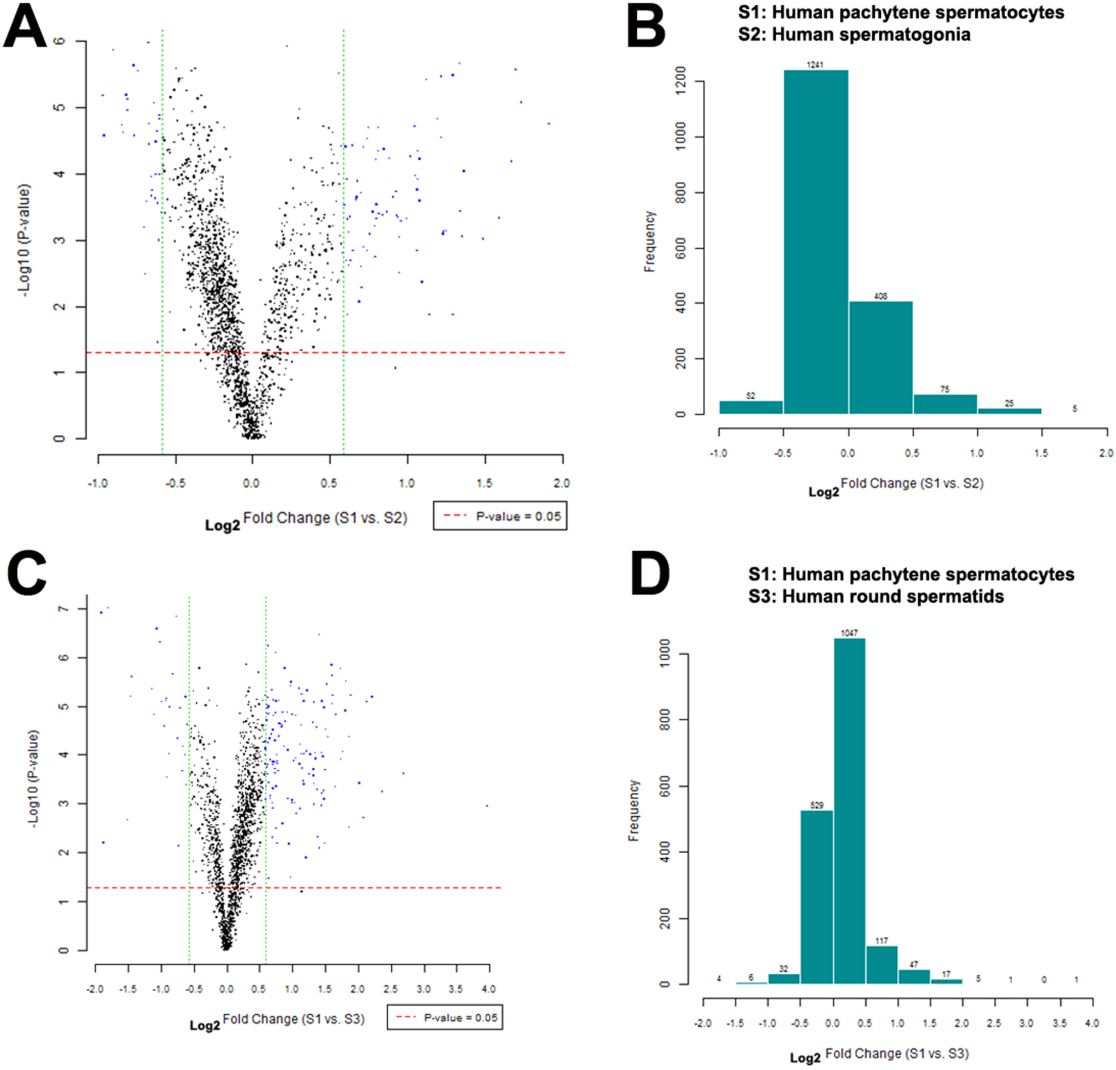
Differentially expressed miRNAs among human spermatogonia, pachytene spermatocytes and round spermatids. (A), Scatter plot comparison revealed the patterns of miRNAs between human spermatogonia and pachytene spermatocytes. The log_2_ scales of the expression signal values were plotted for all probes, excluding control and flagged probes. Standard selection criteria to identify differentially expressed miRNAs was established at log_2_|Fold change| ≧ 0.585 and P-value < 0.05 (blue dots). (B), Histogram plot showed fold change distribution of all miRNA probes excluding control and flagged probes between human spermatogonia and pachytene spermatocytes. (C), Scatter plot comparison of miRNA expression patterns between human pachytene spermatocytes and round spermatids. Standard selection criteria to identify differentially expressed miRNAs was established at log_2_|Fold change| ≧0.585 and P-value < 0.05 (blue dots). (D), Histogram plot revealed fold change distribution of all miRNA probes excluding control and flagged probes between human pachytene spermatocytes and round spermatids. The data shown in (A–D) were representatives from two independent experiments of twenty patients.

Scatter plot comparison was used to show the patterns of miRNAs among human spermatogonia, pachytene spermatocytes and round spermatids. The dotted line in red and green represented the cut-off, a measurement of fold change on the x-axis versus a measure of significance (negative logarithm of the P-value) on the y-axis. The log_2_ scales of the expression signal values were plotted for all miRNA probes excluding control and flagged probes between human spermatogonia and pachytene spermatocytes (Fig. 5A) as well as between human pachytene spermatocytes and round spermatids (Fig. 5C). Histogram plots showed fold change distribution of miRNA probes excluding control and flagged probes between human spermatogonia and pachytene spermatocytes (Fig. 5B) as well as between human pachytene spermatocytes and round spermatids (Fig. 5D).

**Figure 6:**
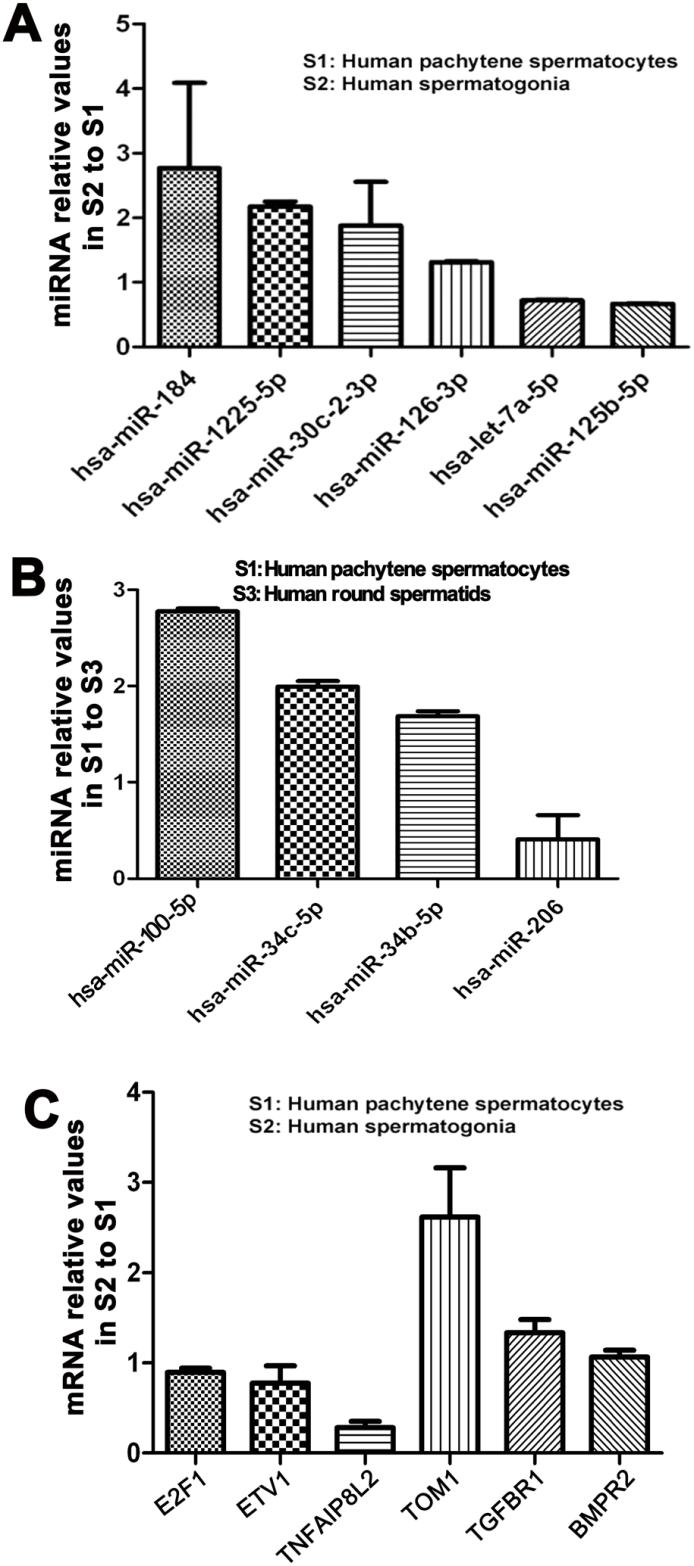
Distinct expression patterns of miRNAs and their targeting genes in human spermatogonia, pachytene spermatocytes and round spermatids. (A), Real time PCR showed that the expression of human miR-184, miR-1225-5p and miR-30c-2-3p was statistically higher in human spermatogonia than pachytene spermatocytes. In contrast, the levels of let-7a-5p, miR-125b-5p, and miR-126-3p were statistically lower in human spermatogonia than pachytene spermatocytes. (B), Real time PCR revealed that miR-100-5p, miR-34c-5p and miR-34b-5p were statistically expressed at higher levels in human pachytene spermatocytes compared to human round spermatids, and conversely, miR-206 was expressed statistically at a lower level in human pachytene spermatocytes compared with human round spermatids. (C), Real time PCR displayed that the transcripts of E2F1, ETV1 and TNFAIP8L2 were expressed statistically at lower levels in human spermatogonia compared to pachytene spermatocytes, which was inversely correlated with the expression levels of human miR-184, miR-1225-5p and miR-30c-2-3p, as shown by miRNA microarrays and real-time PCR analyses. On the contrary, mRNA of TOM1, TGFBR1 and BMPR2 was presented at higher levels in human spermatogonia compared with pachytene spermatocytes, which was inversely correlated with the expression of human miR-126-3p, let-7a-5p and miR-125b-5p, as indicated by miRNA microarrays and real time PCR analyses. The data shown in A–C were presented as mean ± SEM from three independent experiments of nine patients.

In order to verify the data of miRNA microarrays, we further performed real time -PCR for a number of differentially expressed miRNAs among human spermatogonia, pachytene spermatocytes and round spermatids. Differentially expressed miRNAs identified by miRNA microarrays were randomly chosen for real time-PCR assays based on their fold changes in these cell populations. Real time PCR revealed that the expression of human miR-184, miR-1225-5p and miR-30c-2-3p was up-regulated in human spermatogonia compared to pachytene spermatocytes, whereas miR-126-3p, let-7a-5p and miR-125b-5p were down-regulated in human spermatogonia compared with pachytene spermatocytes (Fig. 6A), which was completely consistent with our miRNA microarray data. Likewise, miR-100-5p, miR-34c-5p and miR-34b-5p were up-regulated in human pachytene spermatocytes compared with human round spermatids, whereas miR-206 was down-regulated in human pachytene spermatocytes compared to human round spermatids (Fig. 6B), which was fully consistent with their expression patterns of these miRNAs by our miRNA microarrays. Together, these results confirmed the quality and authenticity of our miRNA microarray data.

### Identification of novel binding targets of differentially expressed miRNAs among human spermatogonia, pachytene spermatocytes and round spermatids

**Figure 7:**
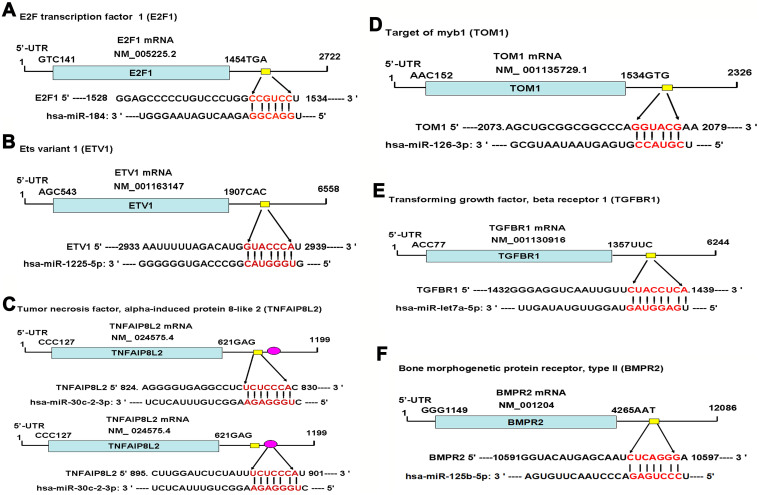
Predicted targets and binding sites of the differentially expressed miRNAs between human spermatogonia and pachytene spermatocytes.

To gain novel insights into molecular mechanisms of differentially expressed miRNAs among human spermatogonia, pachytene spermatocytes and round spermatids, we identified the binding targets of these miRNAs. Using different miRNA software programs, including TargetScan, miRbase, and miRDB^5^, we predicted the targeting genes of human miR-1225-5p, miR-184, miR-30c-2-3p, let-7a-5p, miR-125b-5p and miR-126-3p. Specifically, E2F transcription factor 1 (E2F1) was a potential binding target for human miR-184 (Fig. 7A), while Ets variant 1 (ETV1) was a target of miR-1225-5p (Fig. 7B). We also identified other targets for miRNAs, e.g., tumor necrosis factor alpha-induced protein 8-like 2 (TNFAIP8L2) for miR-30c-2-3p (Fig. 7C), target of myb1 (TOM1) for miR-126-3p (Fig. 7D), transforming growth factor, beta receptor 1 (TGFBR1) for let-7a-5p (Fig. 7E), and bone morphogenetic protein receptor, type II (BMPR2) as a target for miR-125b-5p (Fig. 7F). Real-time PCR further revealed that the transcripts of E2F1, ETV1 and TNFAIP8L2 were down-regulated in human spermatogonia compared to human pachytene spermatocytes (Fig. 6C). In contrast, mRNA of TOM1, TGFBR1 and BMPR2 was up-regulated in human spermatogonia compared with human pachytene spermatocytes (Fig. 6C).

## Discussion

Although much progress has been achieved in understanding the mechanisms of spermatogenesis in rodents, very little is known about the isolation and regulation of male germ cells in humans. Spermatogenesis includes mitosis of spermatogonia, meiosis of spermatocytes and spermiogenesis of round spermatids. To separate human spermatogonia, spermatocytes and round spermatids with a high purity and viability from testis tissues is critical for their basic research and clinical applications. However, information on separation of male germ cells in humans is rather limited. One major reason for so little progress on isolation and characterization of human spermatogenic cell has been the difficulty in gaining access to sufficient quantities of human testis tissues for research purposes. We obtained testicular tissues from OA patients undergoing the testicular biopsy and testicular sperm extraction (TESE) from Ren Ji Hospital affiliated to Shanghai Jiao Tong University School of Medicine, which allowed us to work efficiently on isolation and mechanism studies. It has been reported that STA-PUT approach can be used to isolate spermatogenic cells by a linear BSA gradient and sedimentation velocity at unit gravity based on size and mass of the cells^2^,^26^,^27^,^28^,^29^. Certain techniques have been applied to separate these subtypes of male germ cells, including STA-PUT, FACS, MACS and elutriation^2^,^26^,^27^,^28^,^29^,^30^,^31^. The STA-PUT method has several advantages over FACS, MACS and elutriation for separating spermatogenic cell types. First of all, STA-PUT apparatus assembly is relatively simple since it requires only several pieces of specialized glassware. Thus, it costs less compared to a cell sorter or an elutriator. Secondly, the yield of the isolated cells by STA-PUT approach is higher than FACS in a comparable time frame. Thirdly, a major advantage of the STA-PUT method over FACS or MACS is the ability to obtain more viable cells for subsequent culture and other studies. FACS requires DNA or other types of staining, and thus it might be not an ideal choice for obtaining a higher cell activity. Finally, STA-PUT method is able to isolate subtypes of spermatocytes and round spermatids based on unit gravity and morphological features of these cells. Although MACS has been employed for the enrichment of spermatogonia from a mixture of male germ cells, it is unsuitable for separating spermatocytes or round spermatids due to lacking of suitable markers for these cells.

Here we have obtained highly enriched populations of human spermatogonia, pachytene spermatocytes and round spermatids with high purities and viabilities by the STA-PUT method using sedimentation velocity of unit gravity at 2–4% BSA gradient. Freshly isolated human spermatogonia had the following morphological features: (i) they were spherical in outline; (ii) their nuclei were round or ovoid, and their diameter ranged from 9 to 12 μm; and (iii) they had a high ratio of nucleus to cytoplasm, which indicates that these cells remain an undifferentiated status. The purity of isolated human spermatogonia was around 90%, as evidenced by our observations that 90% of these cells expressed GPR125, GFRA1, UCHL1 and MAGEA4, specific markers for human spermatogonia including SSCs^1^, and that GPR125, GFRA1, PLZF, UCHL1 and RET transcripts were detected in these cells. The contaminating cells in human spermatogonial fraction might be secondary spermatocytes due to their similar size and gravity. Freshly isolated human pachytene spermatocytes possessed the following morphological characteristics: (i) their diameter ranged from 14 to 16 μm, and (ii) they had condensed chromatin. A higher purity of isolated human pachytene spermatocytes was achieved, as demonstrated by our immunocytochemistry and meiosis spread assays showing that 92% of these cells were positive for SCP3 and CREST, hallmarks for pachytene spermatocytes^18^,^19^. The purity was also verified by our findings that SCP1, SCP3 and Tesmin, markers for meiotic prophase of spermatocytes^20^,^21^,^22^,^23^,^24^, were present in the isolated human pachytene spermatocytes. Certain subpopulations of human spermatogonia and diplotene spermatocytes might exist in human pachytene spermatocyte fraction since they have similar gravity. Freshly isolated human round spermatids had unique morphological traits: (i) they were smaller male germ cells with a diameter ranging from 6 to 8 μm; and (ii) they had round nuclei. The purity of isolated human round spermatids was about 95%, since 95% of these cells expressed Acrosin and Protamine 2 as well as the transcripts of ACR, TNP1, PRM1 and PRM2, hallmarks for haploid spermatids^25^. Human enlongated spermatids might remain in human round spermatid fraction because of their similar gravity. To improve the purity of isolated human round spermatids, 20 μm filter meshes may be used to remove enlongated spermatids.

MiRNAs are a class of short non-coding RNA that plays important roles in regulating cellular proliferation^6^, differentiation^7^,^8^, and apoptosis^9^. In rodent reproductive system, the X chromosome-linked miRNAs are essential in the process of spermatogenesis^3^. Increasing evidence has highlighted that miRNAs may be critical players in regulating spermatogenesis^10^,^32^,^33^. It has been shown that miRNA-21 is important in maintaining mouse Thy^+^ spermatogonial population^11^, while miR-17-92 cluster and miR-106b-25 cluster, as well as miR-146 and let-7 have been reported to regulate the differentiation of mouse SSCs^33^,^34^,^35^. We explored the expression, function, and targets of miRNA-20 and miRNA-106a in mouse SSCs^12^. The global expression of miRNAs have been found in murine testis; however, the expression of miRNAs in specific germ cell populations of human testes remains unclear^10^,^36^,^37^. Although miRNAs are highly conserved between animals and humans, their expression patterns, roles and targets of these miRNAs might be distinct. To obtain global expression profiles of miRNAs in male germ cells is a prerequisite for a thorough understanding of their roles and binding targets in regulating spermatogenesis. In the current study, we revealed, using miRNA microarrays, that 559 miRNAs were differentially expressed among human spermatogonia, pachytene spermatocytes and round spermatids. In total, 110 miRNAs were differentially expressed between human spermatogonia and pachytene spermatocytes, reflecting potentially important roles of these miRNAs in regulating human male meiosis and mitosis. Previous studies have reported that miR-184 plays an important role in the balance between proliferation and differentiation of adult neural stem/progenitor cell and in Drosophila female germline development^38^,^39^. In line with these observations, we found a higher expression of miR-184 in human spermatogonia compared to pachytene spermatocytes, suggesting miR-184 may be involved in the self-renewal of human SSCs. We also identified E2F1 as a target for miR-184 using various kinds of softwares. E2F1 has recently been shown as a target of miRNA-383 in mouse spermatogenesis^40^. Members of miR-125 family (hsa-miR-125b-1-3p and hsa-miR-125b-5p) and let-7 family (e.g., has-let-7a-5p, has-let-7b-5p, has-let-7c has-let-7d-5p, has-7e-5p, has-let-7f-5p and has-let-7g-5p) were also differentially expressed between human spermatogonia and pachytene spermatocytes. It has been found that let-7 family miRNAs play important roles in promoting the differentiation of stem cells and inhibiting cell proliferation in tumor cells^35^,^41^. Moreover, 173 miRNAs were differentially expressed between human pachytene spermatocytes and round spermatids, suggesting that these miRNAs might be involved in human male meiosis and spermiogenesis. As examples, we found that miR-34b-5p and miR-34c-5p were up-regulated in human pachytene spermatocytes compared to round spermatids, implicating that these miRNAs might play roles in regulating the meiosis of spermatocytes. Our findings are consistent with recent report demonstrating that miR-34b/c is required for mouse normal spermatogenesis^42^.

We also predicted a number of binding targets for differentially expressed miRNAs among human spermatogonia, pachytene spermatocytes and round spermatids. Interestingly, we found that there was a distinct expression in miRNA targets, including E2F1, ETV1, TNFAIP8L2, TOM1, TGFBR1 and BMPR2, between human human spermatogonia and pachytene spermatocytes, which verified these genes as the targets for their corresponding miRNAs. E2F1 is binding target for miRNA-383 whose down-regulation may be associated with maturation arrest of infertile males^40^,^43^, while BMPR2, a receptor for BMP ligands, has been shown to be expressed in mouse spermatogonia^44^. Additionally, testicular TGFBR1 expression is lower in infant male lambs with prenatal testosterone treatment^45^. Nevertheless, the expression and potential of other miRNA targets, including ETV1, TNFAIP8L2 and TOM1, in male germ cells and spermatogenesis remain to be defined. The miRNA targets identified by us could shed novel insights into the molecular mechanism of miRNAs in regulating normal and aberrant spermatogenesis.

In summary, we have separated human spermatogonia, pachytene spermatocytes and round spermatids from adult testis tissues with high purities and viabilities, which could offer essential cell sources to probe molecular mechanisms controlling mitosis, meiosis and spermiogenesis. We unveiled global distinct miRNA profiling among human spermatogonia, pachytene spermatocytes and round spermatids, and we identified their binding targets, which offers novel insights into epigenetic regulatory mechanisms in mediating the process of human spermatogenesis. Since miRNAs are exclusively expressed in male germ cells in the testis, this study may also provide new targets of gene therapy for male infertility.

## Methods

### Procurement of testicular biopsies from obstructive azoospermic (OA) patients with normal spermatogenesis

Testicular biopsies were obtained from 40 OA patients (age ranging from 22 to 35 years old) who underwent microdissection of testicular biopsy and testicular sperm extraction (MD-TESE) at Ren Ji Hospital affiliated to Shanghai Jiao Tong University School of Medicine. Patients with OA were caused by inflammation and vasoligation but not by congenital absence of the vas deferens or other diseases including cancer. Normal spermatogenesis with sperm production was observed in the testis of all OA patients (Supplementary Fig. 1A–1B).

### Ethics statement

This study was approved by the Institutional Ethical Review Committee of Ren Ji Hospital (license number of ethics statement: 2012-01), Shanghai Jiao Tong University School of Medicine, and an informed consent of testis tissues for research only was obtained from all subjects. All experiments were performed in accordance with relevant guidelines and regulations of the Institutional Ethical Review Committee of Ren Ji Hospital.

### Histological examination

Testicular tissues from 5 OA patients were fixed with 4% paraformaldehyde for 3 hours or with Bouin's fixative overnight, and they were embedded in paraffin and sectioned at 5 μm thickness. Human testis sections were stained with hematoxylin and eosin (H&E) and observed under a microscope.

### Isolation of human spermatogonia, pachytene spermatocytes and round spermatids by STA-PUT velocity sedimentation

Isolation of human spermatogenic cells from testicular biopsies (6 grams) of 30 OA patients was performed for eight times using the STA-PUT apparatus. For each isolation, testicular biopsies from 3–4 OA patients were pooled and washed three times aseptically in Dulbecco modified Eagle medium (DMEM) containing antibiotic with penicillin and streptomycin (Gibco). Seminiferous tubules were isolated from human testis biopsies by the first enzymatic digestion comprising 2 mg/ml collagenase IV (Gibico) and 1 μg/μl DNase I (Gibico) in 34°C water bath for 15 min according to the procedure as described previously^1^. Male germ cells were obtained from seminiferous tubules using a second enzymatic digestion with 4 mg/ml collagenase IV, 2.5 mg/ml hyaluronidase (Sigma), 2 mg/ml trypsin (Sigma) and 1 μg/μl Dnase I and followed by differential plating according to the procedure as previously described^1^. With respect to differential plating, Sertoli and myoid cells attached to the culture plates, whereas male germ cells remained in suspension and were collected by centrifuging at 1000 rpm for 5 min.

The STA-PUT method used a linear BSA gradient and velocity sedimentation to purify spermatogenic cells in term of their size, mass, and gravity. Around 5 × 10^6^ male germ cells were resuspended in 25 ml of 0.5% BSA solution and filtered through a 70 μm mesh to remove cell aggregates and a 40 μm mesh to delete most of sperm tails. The STA-PUT method was utilized to separate human pachytene spermatocytes, spermatogonia and round spermatids from human testis tissues. As shown in Fig. 1A, STA-PUT apparatus (two gradient glass chambers, one cell loading chamber, one standard sedimentation chamber, plastic tubing, and baffles) (ProScience Glass Shop Division, Scarborough, Ontario, Canada) was assembled, and a linear gradient was generated from 300 ml of 2% BSA and 300 ml of 4% BSA solutions to the corresponding chambers. Next, 50 ml of 0.5% BSA were loaded to the loading chamber and went into the sedimentation chamber. Twenty-five milliliters of cells' suspension were loaded, and the stirrer started to move under the tube, which made it possible for the cells to go into the sedimentation chamber (~5–10 min). The stirrer started to work under the 2% BSA solution and artery clips were removed so that both 2% and 4% BSA solutions were introduced to the chamber (~30 min). Artery clips were replaced and the stirrers were switched off, and a gradient of BSA was formed after 3 hours of sedimentation in the standard cell sedimentation chamber. In order to collect different cell types, the first fraction collected was designated as 1 with 15 ml of centrifuge tubes and the rest were numbered up to 45. The cells in each fraction were collected by centrifugation at 1000 rpm for 5 min. The cells were resuspended in 0.2 ml cold phosphate buffer solution (PBS). An aliquot of each fraction was examined carefully under a phase-contrast microscope and a DIC microscope to assess cellular integrity and identify cell types. Fractions containing cells of similar size and morphology were pooled according to the procedure as described previously^2^,^3^. Trypan blue exclusion was used to assess the viability of the freshly isolated cells. The pooled samples for each cell type were prepared for immunocytochemical and meiotic spread assays with specific antibodies and extracting RNA for miRNA microarrays.

### Immunocytochemistry

For immunocytochemical staining, the freshly isolated human spermatogonia, pachytene spermatocytes and round spermatids were fixed with 4% paraformaldehyde (PFA) for 30 min, washed three times with cold PBS and permeabilized in 0.4% triton-X 100 (Sigma-Aldrich) for 5 min. After washing with PBS, the cells were blocked in 1% BSA for 30 min and followed by incubation with primary antibodies, including GPR125 (Abcam), GFRA1 (Abcam), UCHL1 (AbD Serotec), MAGEA4 (melanoma antigen family A, 4, a kind gift from Professor Giulio C. Spagnoli, University Hospital of Basel, Switzerland), SCP3 (Abcam), Acrosin (Santa Cruz), or Protamine 2 (Santa Cruz) at a dilution with 1:200 overnight at 4°C. After extensive washes with PBS for 30 min, the cells were incubated with the secondary antibody IgG (Sigma) conjugated with fluorescein isothiocyanate (FITC) or rhodamine at a 1:200 dilution for 1 hour at room temperature. DAPI (4,6-diamidino-2-phenylindole) was used to label the nuclei, and the cells were observed for epifluorescence under a fluorescence microscope (Leica). For the detection of MAGEA4, peroxidase-conjugated goat anti-mouse IgG (Envision detection kit, DAKO) was used as the secondary antibody and examined under a light microscope.

### Meiotic spread assays

Meiotic spread assays were performed to determine the identity of the freshly isolated pachytene spermatocytes from OA patients by STA-PUT velocity sedimentation. Briefly, cells were lysed by a hypotonic solution and spread evenly over slides layered with 1% PFA and 0.15% Triton X-100. Slides were dried for 24 hours at room temperature in a humid chamber. The cells were treated with 0.04% photoflo for 5 min and blocked with 4% normal serum. Double staining was performed in cells incubated with primary antibodies including SCP3 (Abcam) and CREST (Immunovision) overnight at 37°C in a humid chamber. Alexa 555 donkey anti-rabbit (Molecular Probes, Carlsbad, CA) and Alexa 488 goat anti-mouse (Molecular Probes) were used as the secondary antibodies and incubated for 90 min at 37°C. Cells were washed three times with TBS and treated with antifade (vector laboratories), and images were captured with a fluorescence microscope (Leica).

### RNA extraction and reverse transcription-polymerase chain reaction (RT-PCR)

Total RNA was extracted from the freshly isolated human spermatogonia, pachytene spermatocytes, round spermatids, and human testis tissues, using the TRIzol reagent (Invitrogen, Carlsbad, CA, USA), and the quality and concentrations of total RNA were measured by Nanodrop. Reverse transcription (RT) was performed using the First Strand cDNA Synthesis Kit (Thermo Scientific) and PCR of the cDNA was carried out according to the protocol as described previously^16^. The primers of the chosen genes, including GPR125, GFRA1, PLZF, UCHL1, RET, PIWIL2, SCP1, SCP3, Tesmin, ACR (acrosin), TNP1 (transition protein 1), PRM1 (protamine 1), PRM2 (protamine 2) and GAPDH for RT-PCR were designed and listed in Table 1. The PCR reactions started at 94°C for 2 min and were performed as follows: denaturation at 94°C for 30 sec, annealing at 55–60°C for 45 sec, and elongation at 72°C for 45 sec. After 35 cycles, the samples were incubated for an additional 5 min at 72°C. PCR products were separated by electrophoresis on 2% agarose gel and visualized with ethidium bromide. Images were recorded and band intensities were analyzed using chemiluminescence (Chemi-Doc XRS, Bio-Rad, Hercules, CA).

### MiRNA microarrays

Human testis tissues (around 2.0 grams) were pooled from 20 OA patients, and human spermatogonia, pachytene spermatocytes and round spermatids were separated using STA-PUT velocity sedimentation. Total RNA was extracted from these cells using TRIzol (Invitrogen), and DNase I was used to remove potential genomic DNA contamination. The quality of total RNA was checked by gel and electropherograms (Supplementary Fig. 5), and RNA integrity number (RIN) was used to assess RNA quality showing RIN values equaling or over 6.0. NanoSep®100K was used to separate small RNA from large RNA molecules based on the filtrate. MiRNA microarrays were performed twice with two separate pools of the isolated cells from fresh human testicular tissues using miRNA OneArray® Microarrays (Super Biotek Corporation, Shanghai, China), and representative miRNA microarray data confirmed by real time PCR were shown. Hybridization was performed in OneArray Double Chamber according to the procedure as described previously^12^. The data analysis of miRNA profiling, including data filtering, normalization, and statistical calculations, was processed by R version 2.12.1. Raw data in gpr file format contained probe intensities, background value, the detected signals, signal-to-noise ratio data, probe ID, and brief annotation. MiRNA arrays of the compared sample set were normalized together after filtering probes according to flag note from gpr files. The log_2_(Fold change) was calculated by pair-wise combination and error weighted average. The significant differential expressed miRNAs (DE genes) were selected according to log_2_(Fold change) and P-value with criteria: | log_2_(Fold change)|≧0.585 and P-value < 0.05.

Computational analyses were performed to predict potential binding between the 3′ UTR of target genes and miRNAs using three miRNA target prediction algorithms, including TargetScan (version 6.0), miRbase and miRDB. All potential targets of miRNAs were identified and verified by three miRNA target prediction programs in term of the following criteria: targets located in the 3′-UTR region, seed length of at least 7 base pair (bp), and P < 0.05.

### Real time PCR

RNA was extracted from the freshly isolated human spermatogonia, pachytene spermatocytes and round spermatids using TRIzol reagent (Invitrogen). Quantification was performed with a two-step reaction process including RT and PCR. Each RT reaction consisted of 1 μg RNA, 4 μl of miScript HiSpec Buffer, 2 μl of Nucleics Mix and 2 μl of miScript Reverse Transcriptase Mix (Qiagen, Germany), in a total volume of 20 μl. Reactions were performed in a GeneAmp^®^ PCR System 9700 (Applied Biosystems, USA) for 60 min at 37°C, followed by heat inactivation of RT for 5 min at 95°C. The 20 μl RT reaction mix was diluted by 5 times in nuclease-free water and held at −20°C. Primer sequences of miRNAs used for real time PCR were listed in Tables 2 and 3. Real time PCR was performed in triplicate using LightCycler^®^ 480 II Real-time PCR Instrument (Roche, Swiss) with 10 μl PCR reaction mixture including 1 μl of cDNA, 5 μl of 2 × LightCycler^®^ 480 SYBR Green I Master (Roche, Swiss), 0.2 μl of universal primer (Qiagen, Germany), 0.2 μl of miRNA-specific primer (Tables 2 and 3) and 3.6 μl of nuclease-free water. Reactions were incubated in a 384-well optical plate (Roche, Swiss) at 95°C for 10 min, followed by 40 cycles of 95°C for 10 sec, 60°C for 30 sec. Individual samples were run in triplicate. In the end of the PCR cycles, melting curve analysis was performed to validate the specific generation of the expected PCR products. The expression levels of miRNAs were normalized to U6 and calculated using the 2^−ΔΔCt^ method.

Real time PCR was carried out to evaluate the expression of E2F1, ETV1, TNFAIP8L2, TOM1, TGFBR1 and BMPR2 in freshly isolated human spermatogonia and human pachytene spermatocytes pursue to the methods described previously^16^. The primers of these genes were designed and listed in Table 4. The expression levels of these genes were normalized to ACTB (actin, beta) and calculated using the 2^−ΔΔCt^ method.

### Statistical analysis

All the values were presented as mean ± SEM, and statistically significant differences (P < 0.05) among human spermatogonia, pachytene spermatocytes and round spermatids were determined using the analysis of variance (ANOVA) and a 2-tailed *t*-test.

## Supplementary Material

Supplementary Information Supplementary Figures and Tables

## Figures and Tables

**Table 1:** Primer sequences of the genes used for RT-PCR

Genes	Primers (5′-3′)	PCR product (bp)
GPR125	Sense primer: 5′ TACCCTTTGGACTTGGTT 3′ Anti-sense primer: 5′ CTCCATTCGTCCCATTAG 3′	193
GFRA1	Sense primer: 5′CCAAAGGGAACAACTGCCTG 3′ Anti-sense primer: 5′ CGGTTGCAGACATCGTTGGA 3′	121
PLZF	Sense primer: 5′ CGGTTCCTGGATAGTTTGC 3′ Anti-sense primer: 5′ GGGTGGTCGCCTGTATGT 3′	317
UCHL1	Sense primer: 5′ CCAATGTCGGGTAGATGA 3′ Anti-sense primer: 5′ CAAAGTCCCTCCCACAGA 3′	244
RET	Sense primer: 5′ CTCGTTCATCGGGACTTG 3′ Anti-sense primer: 5′ ACCCTGGCTCCTCTTCAC 3′	126
PIWIL2	Sense primer: 5′ CTTTCCGACCATCGTTCA 3′ Anti-sense primer: 5′ TCTTCCAAGCGTCCTACT 3′	431
SCP1	Sense primer:5′ CTGTTGCCCTCATAGACC 3′ Anti-sense primer: 5′ ACACCTGACTGCTGCTTG 3′	159
SCP3	Sense primer: 5′ CTGCCTTTGATCTTGTTGTG 3′ Anti-sense primer: 5′ CAGGAGTATGGTTGAGATG 3′	325
Tesmin	Sense primer: 5′ TCGGTAGTCAACGGGTCT 3′ Anti-sense primer: 5′ TTCTGGGCTTTCTTCATA 3′	378
ACR	Sense primer: 5′ GTGGCTGTTGTACGTGAAGA3′ Anti-sense primer: 5′ GAAGTCGCAGACGAAGGA 3′	212
TNP1	Sense primer: 5′ ACAAGTGGGAGCGGTAA3′ Anti-sense primer: 5′ TAGTCCACCACCAAAGCG 3′	133
PRM1	Sense primer: 5′ CAAGATGTGGCAAGAGGA3′ Anti-sense primer: 5′ CCGGAGCACGTCGAGGTCTA3′	171
PRM2	Sense primer: 5′ ATGCTGCCGCCTGTGGAT3′ Anti-sense primer: 5′ ATGAGGAGGAGCAAGAGC3′	102
GAPDH	Sense primer: 5′-AATCCCATCACCATCTTCC-3′ Anti-sense primer: 5′-CATCACGCCACAGTTTCC-3′	382

**Table 2:** Primer sequences of miRNAs used for real time PCR

MiRNAs	Primer Sequences	Tm (°C)
U6	caaggatgacagccaaattcg	60
miR-1225-5p	gtgggtacggcccagtgggggg	60
miR-30c-2-3p	ctgggagaaggctgtttactct	60
miR-184	tggacggagaactgataagggt	60
Let-7a-5p	tgaggtagtaggttgtatagtt	60
miR-125b-5p	tccctgagaccctaacttgtga	60
miR-126-3p	tcgtaccgtgagtaataatgcg	60

**Table 3:** Primer sequences of miRNAs used for real time PCR

miRNAs	Forward primer	Reverse primer	Tm (°C)
hsa-miR-34c-5p	ACACTCCAGCTGGG+aggcagtgtagttagct	CTCAAGTGTCGTGGAGTCGGCAA	60
hsa-miR-34b-5p	ACACTCCAGCTGGG+taggcagtgtcattagc	CTCAAGTGTCGTGGAGTCGGCAA	60
hsa-miR-100-5p	ACACTCCAGCTGGG+aacccgtagatccgaa	CTCAAGTGTCGTGGAGTCGGCAA	60
hsa-miR-206	ACACTCCAGCTGGG+tggaatgtaaggaagt	CTCAAGTGTCGTGGAGTCGGCAA	60

**Table 4:** Primer sequences of the genes used for real time PCR

Genes	Primers (5′-3′)	PCR product (bp)
E2F1	Sense primer: 5′ GTGTAGGACGGTGAGAGCAC 3′ Anti-sense primer: 5′ TCAAGGGTAGAGGGAGTTGG 3′	157
ETV1	Sense primer: 5′ GAAGGACCCACATACCAACG 3′ Anti-sense primer: 5′ CGACCAGTCCAGGCAATAA 3′	104
BMPR2	Sense primer: 5′ GTAAGCTCTTGCCGTCTTGC 3′ Anti-sense primer: 5′ ATCTCGATGGGAAATTGCAG 3′	105
TGFBR1	Sense primer: 5′ TCAGCTCTGGTTGGTGTCAG 3′ Anti-sense primer: 5′ ATGTGAAGATGGGCAAGACC 3′	132
TOM1	Sense primer: 5′ ATCATCAACGAGACGGAGGA 3′ Anti-sense primer: 5′ GACTGTGAGAGCCAGCATCA 3′	105
TNFAIP8L2	Sense primer: 5′ TGTGACGGACTCAGGAAGC 3′ Anti-sense primer: 5′ CAGGGTTAGGAAGCCCATTA 3′	119
ACTB	Sense primer: 5′ CATGTACGTTGCTATCCAGGC 3′ Anti-sense primer: 5′ CTCCTTAATGTCACGCACGAT 3′	196
