# Supplementary material for: Fractionation of human spermatogenic cells using STA-PUT gravity sedimentation and their miRNA profiling
Source: Sci Rep. 2015 Jan 30;5:8084. doi: 10.1038/srep08084 (PMC5155379; doi:10.1038/srep08084)
Supplement: Supplementary Information — Supplementary Figures and Tables [file srep08084-s1.pdf]

Fractionation of human spermatogenic cells using STA-PUT gravity sedimentation and their miRNA profiling

Yun Liu, Minghui Niu, Chencheng Yao, Yanan Hai, Qingqing Yuan, Yang Liu, Ying Guo, Zheng Li & Zuping He

Supplementary Information

Supplementary Figure 1

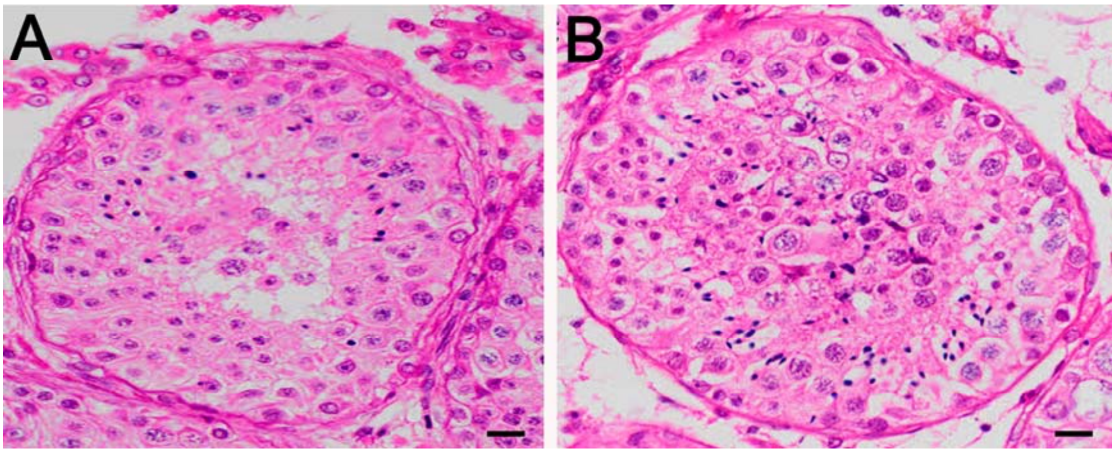

Supplementary Figure 2

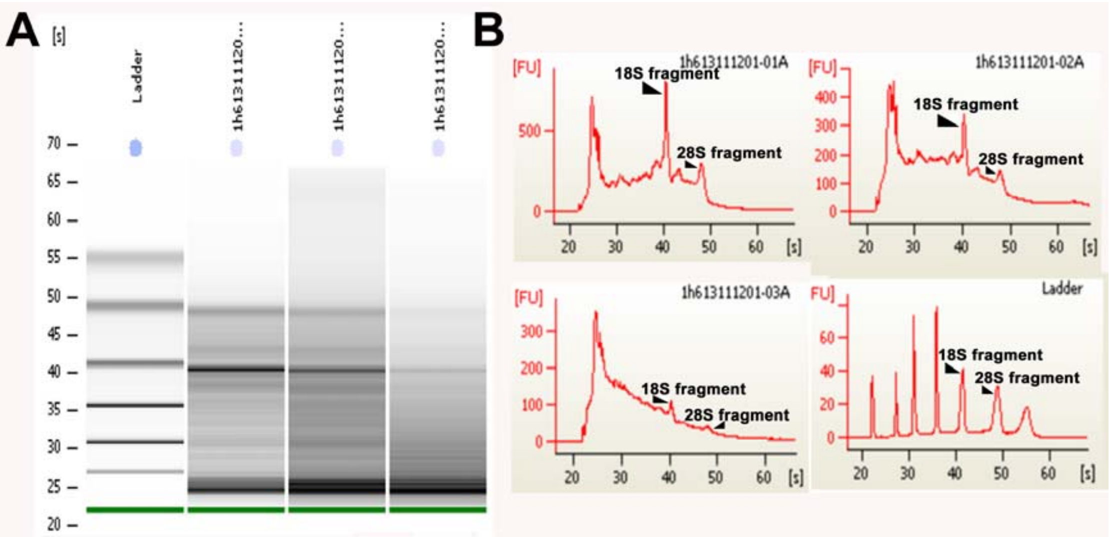

Legends for Supplementary Figures

Supplementary Figure 1. H&E staining showed the morphology of representative testis tissues of OA patients (A, B). Scale bars in A, B = 20 μm.

Supplementary Figure 2. (A-B), Gel imaging (A) and electropherogram (B) assays reflected the quality of total RNA isolated from human spermatogonia, pachytene spermatocytes and round spermatids.

Supplementary Tables

Supplementary Table 1. Up-regulated miRNAs between human spermatogonia and pachytene spermatocytes

		Normalized Intensity			log_2 (Ratio)	P-value (Differentially expressed)
ID	Name	S2	S1	S2/S1	S1/S2	S1/S2
PH_mr_0004621	hsa-miR-4725-3p	6929.750254	3545.08495	1.954748716	-0.96698316	6.65868E-06
PH_mr_0003464	hsa-miR-1908	13560.3136	6974.03186	1.944400868	-0.959325683	2.67413E-05
PH_mr_0001438	hsa-miR-1225-5p	1590.414628	853.2121418	1.864031874	-0.89842653	1.37044E-06
PH_mr_0004266	hsa-miR-3164	554.7676309	309.3181484	1.793517884	-0.842792131	1.8473E-05
PH_mr_0004255	hsa-miR-3153	1297.284133	727.8750482	1.782289606	-0.833731781	2.38004E-05
PH_mr_0003467	hsa-miR-1469	352.4833638	200.0793971	1.761717442	-0.816982552	6.53647E-06
PH_mr_0001970	hsa-miR-30b-3p	1895.657953	1083.18846	1.750072146	-0.807414397	1.11696E-05
PH_mr_0004699	hsa-miR-642b-3p	3931.824738	2248.018514	1.749017952	-0.806545098	7.66176E-06
PH_mr_0004558	hsa-miR-4665-5p	1104.69013	648.5332183	1.703367073	-0.768389367	2.32871E-06
PH_mr_0001371	hsa-miR-1224-5p	4343.660971	2558.486544	1.697746264	-0.763620857	2.68238E-05
PH_mr_0002326	hsa-miR-575	1086.520884	649.6830999	1.672385944	-0.741907823	2.82981E-06
PH_mr_0004211	hsa-miR-2276	926.6315233	570.34127	1.624696602	-0.700170332	0.000644869
PH_mr_0004641	hsa-miR-3158-5p	1449.905795	896.9076423	1.616560866	-0.692927828	0.003256617
PH_mr_0004259	hsa-miR-3156-5p	880.6027679	548.4935197	1.605493477	-0.683016803	0.000262613
PH_mr_0000705	hsa-miR-30c-2-3p	782.4888419	490.9994401	1.593665446	-0.6723488	1.0567E-06
PH_mr_0002420	hsa-miR-30c-1-3p	3567.228544	2241.119224	1.591717435	-0.670584249	3.6139E-05
PH_mr_0001619	hsa-miR-663a	975.0828448	612.8868889	1.590967049	-0.669903956	1.77408E-05
PH_mr_0004669	hsa-miR-3679-5p	13699.61115	8710.353065	1.572796309	-0.653331841	0.000216247
PH_mr_0004045	hsa-miR-3184-5p	641.9800096	408.2079654	1.572678791	-0.65322404	0.000111537
PH_mr_0004748	hsa-miR-4484	6448.870889	4118.875865	1.56568712	-0.646795939	0.000179915
PH_mr_0000512	hsa-miR-320c	1876.277424	1206.225791	1.555494368	-0.637373171	8.5358E-06
PH_mr_0002564	hsa-miR-583	463.9214032	300.1190957	1.54579102	-0.628345291	0.000237474
PH_mr_0005048	hsa-miR-4531	530.5419702	343.8145962	1.543104848	-0.625836091	0.000102604
PH_mr_0008683	hsa-miR-6126	21918.16655	14219.43578	1.541423085	-0.624262902	3.33479E-05
PH_mr_0004213	hsa-miR-711	1832.671235	1195.876856	1.532491598	-0.615879164	2.27569E-05
PH_mr_0003304	hsa-miR-675-5p	862.4335223	563.4419804	1.530651872	-0.614146197	0.035055778
PH_mr_0004818	hsa-miR-4698	1336.04519	873.9100104	1.528813235	-0.612412173	0.000282942
PH_mr_0004850	hsa-miR-371b-5p	499.0486112	327.7162539	1.522807018	-0.606733123	0.001004062
PH_mr_0004837	hsa-miR-4732-5p	4319.43531	2844.80706	1.518357913	-0.602511908	1.50658E-05
PH_mr_0000514	hsa-miR-184	369.4413263	243.7748977	1.515501923	-0.599795683	0.000102329
PH_mr_0001866	hsa-miR-150-3p	794.6016723	524.3460063	1.51541475	-0.599712696	1.32038E-05
PH_mr_0004243	hsa-miR-3138	2415.298376	1605.234703	1.504638774	-0.589417173	0.000384259

Supplementary Table 2. Down-regulated miRNAs between human spermatogonia and pachytene spermatocytes

		Normalized Intensity			$\log_2$ (Ratio)	P-value (Differentially expressed)
**ID**	**Name**	**S2**	**S1**	**S2/S1**	**S1/S2**	**S1/S2**
PH_mr_0004578	hsa-miR-4730	266.4822681	400.1587943	0.665941301	0.586533077	0.001603233
PH_mr_0000485	hsa-miR-449a	654.09284	985.4485249	0.663751402	0.591285092	0.010168286
PH_mr_0004592	hsa-miR-4763-5p	28385.20669	42848.03779	0.662462231	0.594089891	0.000298617
PH_mr_0001101	hsa-miR-99b-5p	232.5663431	353.013649	0.658802694	0.602081639	3.9567E-05
PH_mr_0002598	hsa-miR-634	9227.554176	14051.55306	0.656692832	0.606709387	0.002063493
PH_mr_0004654	hsa-miR-3651	365.8074772	558.8424541	0.654580686	0.611357059	0.000121451
PH_mr_0008662	hsa-miR-6511a-3p	9508.57184	14541.40262	0.65389647	0.61286586	0.00048655
PH_mr_0000446	hsa-miR-22-3p	316.1448726	484.1001505	0.653056753	0.614719721	0.013434835
PH_mr_0001697	hsa-miR-20b-5p	347.6382316	534.6949406	0.650161812	0.621129274	0.000762987
PH_mr_0004792	hsa-miR-4641	1551.65357	2388.304068	0.649688451	0.622180035	0.00240665
PH_mr_0004196	hsa-miR-670	283.4402307	438.1048868	0.646968886	0.628231763	0.000449939
PH_mr_0001699	hsa-miR-17-5p	398.5121192	617.4864153	0.645377954	0.631783799	0.002040824
PH_mr_0000770	hsa-miR-30b-5p	302.8207592	473.7512162	0.639197851	0.645665537	3.75031E-05
PH_mr_0000352	hsa-miR-34a-5p	484.5132148	760.0717328	0.637457221	0.649599566	0.001450411
PH_mr_0000996	hsa-miR-130a-3p	242.2566074	385.2103336	0.628894363	0.66911039	0.000245998
PH_mr_0008719	hsa-miR-98-3p	524.485555	837.1137995	0.626540329	0.67452072	0.000225978
PH_mr_0008606	hsa-miR-1229-5p	758.2631811	1214.274962	0.624457561	0.679324567	0.000130836
PH_mr_0003276	hsa-miR-664a-3p	1603.738741	2586.083702	0.620141854	0.689329834	0.00866003
PH_mr_0002482	hsa-miR-1281	5075.275925	8240.051493	0.615927695	0.699167094	0.000194081
PH_mr_0002947	hsa-miR-19b-3p	485.7244978	789.9686542	0.614865533	0.701657157	0.002549037
PH_mr_0008024	hsa-miR-3184-3p	10087.56513	16431.80796	0.613904761	0.703913237	0.005285395
PH_mr_0001893	hsa-miR-30d-5p	295.553061	485.2500321	0.609073759	0.715311146	0.001268905
PH_mr_0004643	hsa-miR-3173-5p	2185.154599	3589.930332	0.608689973	0.716220493	4.03438E-05
PH_mr_0003167	hsa-miR-107	300.3981932	501.3483744	0.599180547	0.738937309	0.001457314
PH_mr_0001807	hsa-miR-1224-3p	5777.820086	9649.806326	0.59874985	0.739974706	2.95691E-05
PH_mr_0008170	hsa-miR-3676-5p	943.5894858	1585.686716	0.595066778	0.748876518	2.03886E-05
PH_mr_0004647	hsa-miR-3609	262.848419	443.8542948	0.592195281	0.7558551	0.000811521
PH_mr_0000379	hsa-miR-574-5p	7016.962633	12009.36335	0.584290976	0.775241087	0.000382172
PH_mr_0004339	hsa-miR-4290	3477.593599	6012.730848	0.578371739	0.789931034	0.000611943
PH_mr_0002874	hsa-miR-21-5p	248.3130226	430.0557157	0.577397331	0.792363655	0.001809887
PH_mr_0001700	hsa-miR-106a-5p	390.0331379	678.4301397	0.574905381	0.798603561	6.11086E-05
PH_mr_0002893	hsa-miR-125a-5p	686.797482	1195.876856	0.574304518	0.800112183	0.000481501
PH_mr_0001853	hsa-miR-877-3p	4938.400942	8605.71384	0.573851401	0.801250896	0.000291823
PH_mr_0003354	hsa-miR-1258	179.2698895	315.0675564	0.568988732	0.813528012	2.02071E-05
PH_mr_0004911	hsa-miR-4646-3p	4650.115579	8174.508242	0.568855696	0.813865369	4.6105E-05

PH_mr_0004336	hsa-miR-4286	8126.497895	14311.4263	0.567832844	0.816461797	0.000405512
PH_mr_0001891	hsa-miR-30a-5p	259.2145699	464.5521634	0.55798808	0.841693791	0.000422797
PH_mr_0005021	hsa-miR-4455	1757.571687	3163.324262	0.555609081	0.847857915	4.31472E-05
PH_mr_0004646	hsa-miR-3607-5p	245.8904565	448.4538212	0.548307194	0.866943691	0.000376802
PH_mr_0000142	hsa-miR-98-5p	329.4689861	608.2873625	0.541633784	0.884610366	5.87546E-05
PH_mr_0000333	hsa-miR-497-5p	274.9612494	516.2968351	0.532564274	0.908972442	0.00022628
PH_mr_0004085	hsa-miR-3149	2351.100375	4473.039396	0.525615843	0.927919332	0.000188402
PH_mr_0001948	hsa-miR-199a-5p	336.7366843	641.6339287	0.524811219	0.930129534	0.000430942
PH_mr_0000149	hsa-miR-29b-3p	266.4822681	510.5474272	0.521953993	0.938005448	0.000456434
PH_mr_0008666	hsa-miR-6716-3p	937.5330706	1806.463982	0.518987968	0.946227004	0.000904642
PH_mr_0001124	hsa-miR-10b-5p	244.6791735	472.6013346	0.517728486	0.949732396	0.003110353
PH_mr_0003339	hsa-miR-424-5p	666.2056703	1294.766673	0.51453724	0.958652598	0.000526557
PH_mr_0000525	hsa-miR-26b-5p	297.9756271	594.4887834	0.501230024	0.99645526	0.00047356
PH_mr_0001874	hsa-miR-24-3p	459.076271	931.4040901	0.492886252	1.020673354	5.52966E-05
PH_mr_0008021	hsa-miR-5100	4349.717386	8998.973344	0.48335707	1.048838752	1.9564E-05
PH_mr_0004335	hsa-miR-4284	11422.39904	23703.65915	0.481883365	1.053244095	0.000108352
PH_mr_0000205	hsa-let-7e-5p	552.3450649	1153.331237	0.478912776	1.062165171	0.000174487
PH_mr_0002734	hsa-miR-199a-3p,hsa-miR-199b-3p	359.751062	752.0225616	0.478378017	1.063776999	0.001463318
PH_mr_0003406	hsa-miR-1248	311.2997405	651.9828631	0.477466139	1.06652967	4.57372E-05
PH_mr_0000117	hsa-miR-32-3p	1177.367112	2471.095543	0.47645552	1.069586559	0.000121031
PH_mr_0004733	hsa-miR-4454	13113.35016	27707.54686	0.473277199	1.079242677	6.07098E-05
PH_mr_0002298	hsa-miR-27a-3p	605.6415185	1280.968094	0.472799847	1.080698527	0.000256961
PH_mr_0000222	hsa-let-7g-5p	485.7244978	1038.343078	0.467788064	1.096073046	0.004325774
PH_mr_0002737	hsa-miR-29a-3p	421.5264969	917.605511	0.459376597	1.122250735	3.81485E-06
PH_mr_0002203	hsa-miR-27b-3p	399.7234022	883.1090632	0.452631978	1.143589581	0.013514202
PH_mr_0002736	hsa-miR-29c-3p	291.9192119	668.0812054	0.43695169	1.194454312	1.51278E-05
PH_mr_0001589	hsa-miR-16-5p	1317.875944	3067.884089	0.429571622	1.219029405	2.85784E-05
PH_mr_0000017	hsa-miR-100-5p	232.5663431	541.5942302	0.429410673	1.219570044	3.43254E-06
PH_mr_0000207	hsa-let-7d-5p	794.6016723	1866.257825	0.425772721	1.231844575	0.00081694
PH_mr_0000163	hsa-let-7b-5p	1044.125978	2460.746608	0.424312676	1.236800317	0.000755318
PH_mr_0000023	hsa-miR-126-3p	352.4833638	838.2636811	0.420492229	1.249848956	0.000728917
PH_mr_0000042	hsa-miR-125b-5p	781.2775588	1914.552852	0.408073122	1.293100406	0.013305105
PH_mr_0001774	hsa-miR-125b-1-3p	330.6802691	811.8164044	0.40733381	1.295716527	3.30326E-06
PH_mr_0000129	hsa-miR-363-5p	4164.391081	10540.96456	0.395067364	1.339829423	2.1798E-06
PH_mr_0002528	hsa-miR-23b-3p	411.8362326	1044.092486	0.394444207	1.342106846	0.000371313
PH_mr_0000750	hsa-miR-26a-5p	664.9943873	1701.824757	0.390753739	1.355668416	0.000896776
PH_mr_0000169	hsa-let-7c	1057.450091	2724.069493	0.388187634	1.365173934	9.24107E-05
PH_mr_0001128	hsa-miR-99a-5p	239.8340413	673.8306133	0.355926306	1.490349529	0.000971229
PH_mr_0002380	hsa-miR-23a-3p	385.1880058	1162.53029	0.331335888	1.593633621	0.00046493

PH_mr_0002054	hsa-miR-143-3p	771.5872945	2460.746608	0.313558207	1.673194814	6.55836E-05
PH_mr_0000823	hsa-miR-145-5p	892.7155982	2903.451022	0.307467077	1.701496158	2.68582E-06
PH_mr_0000206	hsa-let-7f-5p	1027.168015	3424.347383	0.299960226	1.737156879	8.36704E-06
PH_mr_0000203	hsa-let-7a-5p	1110.746545	4185.568997	0.26537528	1.913894106	1.79242E-05

Supplementary Table 3. Up-regulated miRNAs between human pachytene spermatocytes and round spermatids

		Normalized Intensity			log_2 (Ratio)	P-value (Differentially expressed)
**ID**	**Name**	**S3**	**S1**	**S1/S3**	**S1/S3**	**S1/S3**
PH_mr_0000645	hsa-miR-520a-5p	79.69374239	119.5876856	1.500590662	0.585530486	0.000221888
PH_mr_0001842	hsa-miR-139-5p	91.89909032	137.9857911	1.501492459	0.586397229	5.34137E-05
PH_mr_0000469	hsa-miR-335-5p	118.4636711	178.2316469	1.504525777	0.589308825	7.61292E-05
PH_mr_0000484	hsa-miR-143-5p	119.8995944	180.5314101	1.505688246	0.59042309	7.12402E-05
PH_mr_0004601	hsa-miR-3162-3p	3522.319821	5307.853432	1.506919786	0.591602624	0.000826788
PH_mr_0001239	hsa-miR-191-5p	277.8511559	419.7067813	1.510545385	0.595069531	0.000447765
PH_mr_0000329	hsa-miR-377-3p	95.48889854	144.8850807	1.517297643	0.601504122	4.73226E-05
PH_mr_0002897	hsa-miR-135a-5p	82.56558896	125.3370936	1.518030637	0.602200908	0.000408071
PH_mr_0000913	hsa-miR-424-3p	129.9510574	197.7796339	1.521954787	0.605925501	1.94666E-05
PH_mr_0000433	hsa-miR-296-5p	90.46316704	137.9857911	1.525325673	0.609117306	9.47483E-06
PH_mr_0002986	hsa-miR-136-5p	112.719978	172.4822389	1.530183398	0.613704575	0.000179543
PH_mr_0000010	hsa-miR-7-5p	103.3864766	158.6836598	1.534858958	0.618106089	1.42127E-05
PH_mr_0000812	hsa-miR-92b-3p	269.2356162	413.9573734	1.537528278	0.620612945	0.000157018
PH_mr_0004548	hsa-miR-4634	463.8032215	714.0764691	1.539610844	0.622565737	1.10444E-05
PH_mr_0001853	hsa-miR-877-3p	5589.331392	8605.71384	1.539667849	0.622619153	0.000244143
PH_mr_0008636	hsa-miR-6500-5p	132.1049423	203.5290419	1.540661828	0.623550228	6.68848E-06
PH_mr_0001946	hsa-miR-199b-5p	104.1044383	160.983423	1.546364648	0.628880561	1.47995E-05
PH_mr_0004040	hsa-miR-3178	1536.437916	2376.805252	1.546958212	0.629434226	5.91649E-07
PH_mr_0000647	hsa-miR-145-3p	87.59132046	135.6860279	1.549080745	0.631412346	5.58992E-05
PH_mr_0008677	hsa-miR-6073	94.7709369	147.1848439	1.553058867	0.635112514	0.033031543
PH_mr_0004830	hsa-miR-4716-5p	360.4167449	559.9923356	1.553735623	0.635741042	0.000363533
PH_mr_0004749	hsa-miR-4485	679.1917144	1059.040947	1.559266588	0.640867607	1.02158E-05
PH_mr_0004727	hsa-miR-4445-5p	170.8748711	266.7725295	1.561215689	0.642669866	1.42022E-05
PH_mr_0000770	hsa-miR-30b-5p	301.5438901	473.7512162	1.571085443	0.651761643	4.57301E-05
PH_mr_0000352	hsa-miR-34a-5p	480.3163393	760.0717328	1.582439885	0.662150695	0.000776969
PH_mr_0000965	hsa-miR-106b-5p	206.0549916	326.5663723	1.584850577	0.664346827	0.000124391
PH_mr_0000045	hsa-miR-9-5p	81.84762732	129.93662	1.587542904	0.666795582	0.00069201
PH_mr_0002575	hsa-miR-491-3p	178.7724491	286.3205166	1.601591956	0.679506634	3.76333E-05
PH_mr_0008650	hsa-miR-6723-5p	160.1054464	257.5734768	1.608773983	0.685961656	4.69548E-06
PH_mr_0001599	hsa-miR-126-5p	82.56558896	133.3862648	1.615518843	0.691997578	0.000958578
PH_mr_0004147	hsa-miR-4328	225.4399559	365.6623465	1.621994402	0.69776884	0.000138319
PH_mr_0000014	hsa-miR-31-5p	154.3617533	250.6741872	1.623939751	0.699498109	0.004872711
PH_mr_0002335	hsa-miR-30c-5p	234.0554957	380.6108072	1.62615625	0.701465886	0.000489744
PH_mr_0000566	hsa-miR-151a-5p, hsa-miR-151b	207.4909149	338.0651883	1.62930116	0.704253296	3.10061E-05

PH_mr_0004876	hsa-miR-5096	3388.060994	5526.330935	1.631119081	0.705862111	0.000157449
PH_mr_0001494	hsa-miR-140-3p	153.6437916	250.6741872	1.631528255	0.706223972	0.001208445
PH_mr_0001101	hsa-miR-99b-5p	216.1064546	353.013649	1.633517377	0.707981802	7.50872E-06
PH_mr_0000751	hsa-miR-146b-5p	114.1559013	187.4306996	1.641883578	0.715351833	1.57845E-05
PH_mr_0001183	hsa-miR-518e-3p	77.53985746	127.6368568	1.646080622	0.719034998	0.000137515
PH_mr_0004092	hsa-miR-3182	236.2093806	393.2595047	1.664876745	0.735415375	5.06517E-05
PH_mr_0002876	hsa-miR-425-5p	140.7204821	234.5758449	1.666963057	0.737222132	0.000237383
PH_mr_0004651	hsa-miR-23c	87.59132046	146.0349623	1.667230971	0.737453983	0.0001912
PH_mr_0004277	hsa-miR-3176	360.4167449	602.5379546	1.671781245	0.741386081	8.06511E-07
PH_mr_0000404	hsa-miR-517a-3p, hsa-miR-517b-3p	96.92482183	162.1333046	1.672773821	0.742242389	1.37469E-05
PH_mr_0001124	hsa-miR-10b-5p	280.7230025	472.6013346	1.683514819	0.75147642	0.003210357
PH_mr_0000436	hsa-miR-92a-3p	298.6720436	503.6481376	1.686291531	0.753853975	0.000442588
PH_mr_0004563	hsa-miR-4687-5p	6499.706755	10969.87039	1.68774851	0.755099946	7.96494E-06
PH_mr_0000581	hsa-miR-181a-5p	127.0792108	215.0278578	1.692077378	0.758795544	9.9919E-05
PH_mr_0000398	hsa-miR-517c-3p	93.33501361	158.6836598	1.700151461	0.765663278	0.000151057
PH_mr_0000191	hsa-miR-519b-3p	79.69374239	135.6860279	1.702593251	0.767733817	0.000133811
PH_mr_0001737	hsa-miR-28-5p	155.0797149	264.4727663	1.705398842	0.770109183	0.006429528
PH_mr_0000747	hsa-miR-186-5p	75.38597253	129.93662	1.72361801	0.785440079	0.000212434
PH_mr_0001189	hsa-miR-374a-5p	91.89909032	158.6836598	1.726716328	0.788031091	4.20414E-05
PH_mr_0004057	hsa-miR-4321	113.4379396	196.6297524	1.73336851	0.7935784	7.69414E-06
PH_mr_0001355	hsa-miR-127-3p	98.36074511	171.3323573	1.741877383	0.800643071	2.37202E-05
PH_mr_0002107	hsa-miR-153	79.69374239	139.1356727	1.74587952	0.803954005	2.21498E-05
PH_mr_0004038	hsa-miR-1193	269.2356162	473.7512162	1.759615696	0.815260376	2.3489E-05
PH_mr_0000511	hsa-miR-520b,hsa -miR-520c-3p	96.20686018	171.3323573	1.780874638	0.832585964	2.75165E-05
PH_mr_0004728	hsa-miR-4446-5p	313.0312764	561.1422172	1.792607511	0.842059647	2.51962E-05
PH_mr_0008666	hsa-miR-6716-3p	1005.1463	1806.463982	1.797214974	0.845762987	0.002535634
PH_mr_0004654	hsa-miR-3651	310.8773915	558.8424541	1.797629771	0.846095923	1.36211E-05
PH_mr_0000399	hsa-miR-519a-3p	94.05297525	172.4822389	1.83388392	0.874902323	6.66992E-05
PH_mr_0001673	hsa-miR-18b-5p	82.56558896	151.7843702	1.838349028	0.878410702	1.68333E-06
PH_mr_0001084	hsa-miR-660-5p	116.3097862	216.1777394	1.858637579	0.894245483	2.06457E-05
PH_mr_0000341	hsa-miR-148a-3p	87.59132046	165.5829493	1.890403621	0.918694298	1.34186E-05
PH_mr_0000663	hsa-miR-1294	312.3133148	594.4887834	1.903501245	0.928655513	0.000784781
PH_mr_0000557	hsa-miR-376a-3p	101.9505533	194.3299892	1.906120004	0.930638951	7.9326E-05
PH_mr_0001122	hsa-miR-10a-5p	110.566093	212.7280946	1.923990337	0.944101554	0.006587085
PH_mr_0000050	hsa-miR-668	381.2376325	746.2731537	1.95750128	0.969013251	3.81075E-06
PH_mr_0000222	hsa-let-7g-5p	529.8556926	1038.343078	1.959671459	0.970611805	0.031580967
PH_mr_0000177	hsa-miR-520f	84.71947389	166.7328309	1.968057913	0.976772674	3.24924E-06
PH_mr_0000023	hsa-miR-126-3p	421.4434845	838.2636811	1.989029874	0.992064945	0.000832127

PH_mr_0002947	hsa-miR-19b-3p	397.0327887	789.9686542	1.989681147	0.992537252	0.00094915
PH_mr_0001683	hsa-miR-140-5p	73.2320876	146.0349623	1.994139005	0.995765979	1.48274E-05
PH_mr_0002984	hsa-miR-142-3p	68.2063561	137.9857911	2.023063524	1.016541621	0.000131319
PH_mr_0000150	hsa-let-7i-5p	198.8753752	408.2079654	2.052581749	1.037439682	0.000948346
PH_mr_0001528	hsa-miR-1246	12449.45489	25743.5491	2.067845486	1.048128388	0.000116651
PH_mr_0002946	hsa-miR-19a-3p	206.0549916	426.6060709	2.070350578	1.049875084	1.55166E-05
PH_mr_0003348	hsa-miR-101-3p	92.61705197	193.1801076	2.085794176	1.060596801	0.001564197
PH_mr_0000849	hsa-miR-144-3p	94.05297525	201.2292787	2.13953124	1.097294744	4.35566E-06
PH_mr_0001697	hsa-miR-20b-5p	247.6967669	534.6949406	2.15866742	1.110140988	0.000152156
PH_mr_0003267	hsa-miR-15b-5p	260.6200765	565.7417436	2.170752734	1.118195401	0.001245694
PH_mr_0000996	hsa-miR-130a-3p	177.3365259	385.2103336	2.172199617	1.119156688	6.13124E-05
PH_mr_0002893	hsa-miR-125a-5p	546.3688104	1195.876856	2.188772188	1.130121803	9.53199E-05
PH_mr_0000142	hsa-miR-98-5p	277.8511559	608.2873625	2.189256189	1.13044079	2.48599E-05
PH_mr_0000555	hsa-miR-376c-3p	99.7966684	219.6273842	2.20074866	1.137994389	5.73515E-06
PH_mr_0000333	hsa-miR-497-5p	232.6195724	516.2968351	2.219490088	1.150228266	0.000186824
PH_mr_0001699	hsa-miR-17-5p	275.697271	617.4864153	2.239726252	1.163322411	0.000342179
PH_mr_0001696	hsa-miR-20a-5p	255.594345	576.0906779	2.253925759	1.172439996	7.89684E-06
PH_mr_0003167	hsa-miR-107	221.8501477	501.3483744	2.259851434	1.176227931	0.000395999
PH_mr_0001893	hsa-miR-30d-5p	214.6705313	485.2500321	2.260440821	1.176604148	9.55207E-05
PH_mr_0002203	hsa-miR-27b-3p	382.6735558	883.1090632	2.307734751	1.206477412	0.012505792
PH_mr_0002951	hsa-miR-103a-3p	200.3112984	465.702045	2.32489155	1.21716342	8.33421E-05
PH_mr_0008170	hsa-miR-3676-5p	679.9096761	1585.686716	2.332202015	1.22169276	4.81649E-06
PH_mr_0001728	hsa-miR-18a-5p	109.8481314	262.1730031	2.386686053	1.255008805	0.000243943
PH_mr_0000169	hsa-let-7c	1128.635703	2724.069493	2.413595003	1.271183615	9.71779E-05
PH_mr_0004335	hsa-miR-4284	9535.248583	23703.65915	2.485898395	1.313767331	2.18408E-05
PH_mr_0004647	hsa-miR-3609	178.0544875	443.8542948	2.492800384	1.317767361	0.000197019
PH_mr_0001119	hsa-miR-15a-5p	180.2083724	450.7535843	2.501291024	1.322672924	0.000267964
PH_mr_0002528	hsa-miR-23b-3p	416.417753	1044.092486	2.507319821	1.326146031	0.000385325
PH_mr_0005109	hsa-miR-3607-3p	89.74520539	225.3767922	2.511296188	1.328432193	0.001036906
PH_mr_0001890	hsa-miR-30e-5p	134.9767889	339.2150698	2.513136315	1.329488926	8.33815E-06
PH_mr_0001948	hsa-miR-199a-5p	252.0045367	641.6339287	2.546120546	1.348300725	9.15023E-05
PH_mr_0001891	hsa-miR-30a-5p	182.3622574	464.5521634	2.547413978	1.34903343	0.000119866
PH_mr_0004646	hsa-miR-3607-5p	173.7467176	448.4538212	2.581077946	1.36797371	2.70429E-05
PH_mr_0000446	hsa-miR-22-3p	186.6700272	484.1001505	2.593346976	1.374815244	0.000509134
PH_mr_0004578	hsa-miR-4730	152.92583	400.1587943	2.61668545	1.387740509	0.004677509
PH_mr_0000207	hsa-let-7d-5p	702.8844486	1866.257825	2.655141721	1.408788869	0.007956329
PH_mr_0000017	hsa-miR-100-5p	203.9011067	541.5942302	2.656161308	1.409342764	3.45543E-07
PH_mr_0000042	hsa-miR-125b-5p	705.7562952	1914.552852	2.712767658	1.439765489	0.001069528
PH_mr_0002247	hsa-miR-195-5p	382.6735558	1039.49296	2.716396114	1.441693873	9.15766E-05
PH_mr_0001700	hsa-miR-106a-5p	248.4147285	678.4301397	2.731038307	1.44944955	7.94052E-06

PH_mr_0000163	hsa-let-7b-5p	900.3239005	2460.746608	2.733179256	1.450580082	0.000605409
PH_mr_0000525	hsa-miR-26b-5p	216.1064546	594.4887834	2.750907115	1.459907427	0.000107313
PH_mr_0000149	hsa-miR-29b-3p	185.2341039	510.5474272	2.756228018	1.462695245	0.000108654
PH_mr_0002734	hsa-miR-199a-3p, hsa-miR-199b-3p	270.6715395	752.0225616	2.77835846	1.474232746	0.000531162
PH_mr_0001128	hsa-miR-99a-5p	241.2351121	673.8306133	2.793252638	1.481946065	0.000804957
PH_mr_0000206	hsa-let-7f-5p	1223.40664	3424.347383	2.799026318	1.484925052	1.06598E-05
PH_mr_0002380	hsa-miR-23a-3p	412.8279448	1162.53029	2.816016466	1.49365577	0.00644988
PH_mr_0002874	hsa-miR-21-5p	152.2078683	430.0557157	2.825449961	1.498480639	0.000202768
PH_mr_0004725	hsa-miR-4443	4302.744127	12194.49429	2.834120257	1.502900976	3.01528E-05
PH_mr_0004730	hsa-miR-4448	1328.22904	3818.756769	2.875073993	1.523599086	3.61317E-06
PH_mr_0000205	hsa-let-7e-5p	397.0327887	1153.331237	2.90487655	1.538476854	4.23722E-05
PH_mr_0002737	hsa-miR-29a-3p	302.9798134	917.605511	3.028602799	1.598652381	1.43882E-06
PH_mr_0001874	hsa-miR-24-3p	306.5696216	931.4040901	3.038148676	1.603192472	9.04571E-06
PH_mr_0000823	hsa-miR-145-5p	952.7351005	2903.451022	3.047490347	1.607621651	2.39556E-06
PH_mr_0002736	hsa-miR-29c-3p	218.9783012	668.0812054	3.050901399	1.609235555	6.13565E-06
PH_mr_0000485	hsa-miR-449a	313.0312764	985.4485249	3.148083272	1.654473703	0.00044402
PH_mr_0001107	hsa-miR-34b-5p	501.1372269	1585.686716	3.164176659	1.661830149	1.50944E-05
PH_mr_0003339	hsa-miR-424-5p	389.8531722	1294.766673	3.321164904	1.731689358	0.000121467
PH_mr_0004549	hsa-miR-4638-5p	1214.7911	4048.733088	3.33286364	1.736762292	1.72261E-06
PH_mr_0002170	hsa-miR-34c-5p	437.2386407	1486.796899	3.400424302	1.765714776	8.65895E-05
PH_mr_0000203	hsa-let-7a-5p	1197.560021	4185.568997	3.495080768	1.805325795	1.24418E-05
PH_mr_0001589	hsa-miR-16-5p	873.7593197	3067.884089	3.51113175	1.811936132	3.13207E-06
PH_mr_0002298	hsa-miR-27a-3p	353.2371284	1280.968094	3.626368779	1.858525646	0.002984543
PH_mr_0002054	hsa-miR-143-3p	678.4737528	2460.746608	3.626885489	1.858731196	4.24883E-05
PH_mr_0003406	hsa-miR-1248	176.6185642	651.9828631	3.691474143	1.884197053	6.12653E-06
PH_mr_0000750	hsa-miR-26a-5p	420.0075612	1701.824757	4.051890762	2.01859528	0.000377033
PH_mr_0002253	hsa-miR-449b-5p	313.0312764	1328.11324	4.242749334	2.084999445	0.001911233
PH_mr_0004336	hsa-miR-4286	3273.187131	14311.4263	4.37232145	2.128399471	8.1742E-06
PH_mr_0004733	hsa-miR-4454	5977.030679	27707.54686	4.635670845	2.212778132	6.49485E-06
PH_mr_0008021	hsa-miR-5100	1748.954563	8998.973344	5.145344274	2.363267611	0.000563224
PH_mr_0000129	hsa-miR-363-5p	1634.798661	10540.96456	6.447867134	2.688822016	0.000241909
PH_mr_0000212	hsa-miR-451a	104.8223999	1631.68198	15.56615744	3.960340949	0.001102745

Supplementary Table 4. Down-regulated miRNAs between human pachytene spermatocytes and round spermatids

		Normalized Intensity		log_2 (Ratio)		P-value (Differentially expressed)
ID	Name	S3	S1	S1/S3	S1/S3	S1/S3
PH_mr_0008641	hsa-miR-6514-5p	1825.776459	486.3999137	0.266407156	-1.90829526	1.25122E-07
PH_mr_0000903	hsa-miR-1	777.5524595	211.5782131	0.272107959	-1.877748938	0.006184642
PH_mr_0008048	hsa-miR-5194	6587.298076	1889.255457	0.286802788	-1.801869044	9.93142E-08
PH_mr_0008715	hsa-miR-6128	2011.728524	703.7275347	0.349812376	-1.515346764	0.002114741
PH_mr_0001363	hsa-miR-206	1522.796645	554.2429277	0.363963849	-1.458132936	6.30378E-06
PH_mr_0004084	hsa-miR-3146	1400.743166	513.9970719	0.366945979	-1.446360408	2.50603E-06
PH_mr_0004235	hsa-miR-3127-5p	2781.383406	1266.019634	0.455176237	-1.135502851	4.45491E-06
PH_mr_0004988	hsa-miR-3673	274.2613477	127.6368568	0.46538405	-1.103506328	1.51156E-05
PH_mr_0004287	hsa-miR-3198	2021.779987	964.7506563	0.477178853	-1.067397984	2.665E-07
PH_mr_0004255	hsa-miR-3153	1473.975253	727.8750482	0.493817686	-1.017949589	4.94348E-07
PH_mr_0004819	hsa-miR-4700-5p	4933.832412	2473.395306	0.501313198	-0.996215878	8.17555E-06
PH_mr_0001373	hsa-miR-135a-3p	863.7078567	446.154058	0.516556674	-0.953001451	2.62181E-05
PH_mr_0001178	hsa-miR-1243	492.5216872	258.7233584	0.525303484	-0.928776941	7.83622E-06
PH_mr_0005048	hsa-miR-4531	651.1912103	343.8145962	0.527977944	-0.921450433	6.97215E-06
PH_mr_0008716	hsa-miR-6130	4401.822834	2337.709278	0.531077548	-0.913005556	0.000284536
PH_mr_0005148	hsa-miR-3145-5p	6427.910591	3487.590871	0.54256991	-0.882119055	9.39715E-05
PH_mr_0000514	hsa-miR-184	445.1362188	243.7748977	0.547641121	-0.868697317	1.04126E-05
PH_mr_0004266	hsa-miR-3164	549.9586187	309.3181484	0.562438951	-0.830231586	2.25514E-06
PH_mr_0000705	hsa-miR-30c-2-3p	863.7078567	490.9994401	0.568478608	-0.814822034	2.23874E-06
PH_mr_0004902	hsa-miR-4423-3p	1109.9687	653.1327447	0.58842447	-0.765070851	1.46989E-07
PH_mr_0008057	hsa-miR-5187-5p	1019.505533	605.9875994	0.594393635	-0.750509429	4.59108E-05
PH_mr_0004115	hsa-miR-205-3p	275.697271	165.5829493	0.600596984	-0.735530866	0.007227073
PH_mr_0004903	hsa-miR-4445-3p	1259.304722	769.2707855	0.610869452	-0.711063998	1.10065E-05
PH_mr_0002129	hsa-miR-576-3p	672.7300596	422.0065445	0.627304427	-0.672762351	0.000209194
PH_mr_0008717	hsa-miR-6508-5p	3730.528698	2353.80762	0.630958186	-0.664383695	6.61619E-05
PH_mr_0005266	hsa-miR-4764-5p	1453.154366	933.7038533	0.642535903	-0.638151024	9.94615E-05
PH_mr_0004134	hsa-miR-4306	838.5791992	543.8939933	0.648589893	-0.624621552	6.58737E-06
PH_mr_0004211	hsa-miR-2276	875.195243	570.34127	0.651673183	-0.617779466	0.000404368
PH_mr_0004155	hsa-miR-449b-3p	7330.388377	4836.401979	0.659774316	-0.599955477	2.39574E-05

Supplementary Table 5. Up-regulated miRNAs between human spermatogonia and round spermatids

		Normalized Intensity			log_2 (Ratio)	P-value (Differentially expressed)
**ID**	**Name**	**S2**	**S3**	**S2/S3**	**S3/S2**	**S3/S2**
PH_mr_0001098	hsa-let-7i-3p	139.2975493	81.84762732	1.701913101	-0.767157376	0.000371329
PH_mr_0003348	hsa-miR-101-3p	170.7909082	92.61705197	1.844054681	-0.882881436	0.003820345
PH_mr_0000738	hsa-miR-101-5p	117.4944546	77.53985746	1.515278186	-0.599582679	0.001261331
PH_mr_0002951	hsa-miR-103a-3p	327.04642	200.3112984	1.632690829	-0.707251624	6.54506E-05
PH_mr_0001700	hsa-miR-106a-5p	390.0331379	248.4147285	1.570088618	-0.650845989	0.000138214
PH_mr_0000723	hsa-miR-106b-3p	140.5088323	93.33501361	1.505424672	-0.590170521	0.000338533
PH_mr_0001024	hsa-miR-10b-3p	132.029851	78.2578191	1.687113857	-0.754557339	8.06834E-05
PH_mr_0008672	hsa-miR-1178-5p	138.0862662	88.30928211	1.563666502	-0.644932848	8.34349E-05
PH_mr_0008136	hsa-miR-1185-1-3p	1217.339452	669.8582131	1.817309139	-0.861803854	1.38243E-07
PH_mr_0008111	hsa-miR-1185-2-3p	976.2941278	560.7280433	1.741118782	-0.800014629	7.70699E-06
PH_mr_0004038	hsa-miR-1193	438.4844594	269.2356162	1.628627243	-0.703656441	1.0546E-05
PH_mr_0001371	hsa-miR-1224-5p	4343.660971	2789.998945	1.55686832	-0.638646926	3.28412E-05
PH_mr_0001438	hsa-miR-1225-5p	1590.414628	961.3506402	1.654354365	-0.726268295	4.55105E-06
PH_mr_0008608	hsa-miR-1234-5p	15941.69605	9999.769766	1.594206309	-0.672838343	3.07645E-05
PH_mr_0000922	hsa-miR-124-5p	128.3960019	80.41170403	1.596732758	-0.675122872	0.000435111
PH_mr_0001528	hsa-miR-1246	21737.68538	12449.45489	1.746075276	-0.804115757	0.000549038
PH_mr_0008003	hsa-miR-1247-3p	396.0895531	212.5166464	1.863804835	-0.898250799	4.51027E-06
PH_mr_0003406	hsa-miR-1248	311.2997405	176.6185642	1.762553908	-0.817667383	0.001030004
PH_mr_0001599	hsa-miR-126-5p	124.7621528	82.56558896	1.511067194	-0.595567815	0.004932479
PH_mr_0000585	hsa-miR-1263	136.8749832	82.56558896	1.657772746	-0.72924625	0.000352836
PH_mr_0001355	hsa-miR-127-3p	151.4103796	98.36074511	1.539337461	-0.622309541	0.000767352
PH_mr_0008013	hsa-miR-1271-3p	133.2411341	84.00151225	1.586175421	-0.665552333	0.000105207
PH_mr_0000912	hsa-miR-1278	139.2975493	86.15539718	1.616817446	-0.693156794	0.003342252
PH_mr_0000663	hsa-miR-1294	578.9932917	312.3133148	1.853886031	-0.890552556	0.000297748
PH_mr_0000688	hsa-miR-1303	360.962345	233.337534	1.546953629	-0.629429952	3.22165E-05
PH_mr_0001554	hsa-miR-1307-3p	178.0586064	102.668515	1.734305853	-0.794358347	9.59576E-06
PH_mr_0000844	hsa-miR-132-5p	145.3539644	95.48889854	1.522207991	-0.606165499	0.000256187
PH_mr_0000637	hsa-miR-1323	270.1161172	175.1826409	1.541911435	-0.624719901	9.3567E-05
PH_mr_0002897	hsa-miR-135a-5p	141.7201153	82.56558896	1.716454968	-0.779432008	0.000310093
PH_mr_0001842	hsa-miR-139-5p	146.5652475	91.89909032	1.594849818	-0.673420576	7.53981E-05
PH_mr_0001683	hsa-miR-140-5p	136.8749832	73.2320876	1.869057508	-0.902310959	1.83228E-05
PH_mr_0002984	hsa-miR-142-3p	147.7765305	68.2063561	2.166609374	-1.115439068	9.15098E-06
PH_mr_0000849	hsa-miR-144-3p	201.0729841	94.05297525	2.137869468	-1.096173769	3.23647E-06

PH_mr_0000647	hsa-miR-145-3p	138.0862662	87.59132046	1.57648344	-0.656710016	3.25258E-05
PH_mr_0003467	hsa-miR-1469	352.4833638	202.4651834	1.740957916	-0.79988133	6.4513E-06
PH_mr_0000176	hsa-miR-146a-5p	136.8749832	89.74520539	1.525150927	-0.608952017	0.00074207
PH_mr_0000341	hsa-miR-148a-3p	144.1426814	87.59132046	1.645627451	-0.718637765	0.000162325
PH_mr_0001511	hsa-miR-151a-3p	168.3683421	110.566093	1.522784585	-0.606711871	0.000162312
PH_mr_0002107	hsa-miR-153	132.029851	79.69374239	1.656715409	-0.728325797	8.77176E-05
PH_mr_0001119	hsa-miR-15a-5p	337.9479673	180.2083724	1.875317793	-0.907135097	0.00069457
PH_mr_0003267	hsa-miR-15b-5p	408.2023835	260.6200765	1.566273746	-0.647336382	0.00243892
PH_mr_0001589	hsa-miR-16-5p	1317.875944	873.7593197	1.508282561	-0.592906728	0.002547955
PH_mr_0000747	hsa-miR-186-5p	138.0862662	75.38597253	1.831723616	-0.873201836	2.55348E-06
PH_mr_0001728	hsa-miR-18a-5p	196.227852	109.8481314	1.786355849	-0.837019499	0.000126267
PH_mr_0001673	hsa-miR-18b-5p	145.3539644	82.56558896	1.760466633	-0.815957884	0.000108976
PH_mr_0003464	hsa-miR-1908	13560.3136	7216.950437	1.878953405	-0.909929291	3.04127E-05
PH_mr_0000522	hsa-miR-190a	128.3960019	84.71947389	1.515542956	-0.599834744	0.001414108
PH_mr_0002396	hsa-miR-191-3p	135.6637001	77.53985746	1.749599555	-0.807024759	0.000257463
PH_mr_0003484	hsa-miR-1911-5p	133.2411341	82.56558896	1.613761081	-0.690427002	0.031947738
PH_mr_0003492	hsa-miR-1914-3p	1086.520884	710.7820267	1.528627404	-0.612236799	5.97318E-05
PH_mr_0004003	hsa-miR-1915-3p	558.40148	348.9293586	1.600328165	-0.678367776	8.41503E-05
PH_mr_0002247	hsa-miR-195-5p	742.5165017	382.6735558	1.940339201	-0.95630888	0.000352095
PH_mr_0001946	hsa-miR-199b-5p	161.1006439	104.1044383	1.547490641	-0.629930685	9.63201E-05
PH_mr_0003126	hsa-miR-19b-2-5p	136.8749832	90.46316704	1.513046554	-0.597456378	0.001135072
PH_mr_0001726	hsa-miR-203a	122.3395867	76.10393417	1.607533015	-0.684848367	0.000616041
PH_mr_0001203	hsa-miR-205-5p	132.029851	86.15539718	1.532461753	-0.615851068	3.89728E-06
PH_mr_0001910	hsa-miR-20a-3p	127.1847189	71.79616432	1.77146955	-0.824946667	8.5564E-06
PH_mr_0001696	hsa-miR-20a-5p	387.6105718	255.594345	1.516506838	-0.600752003	0.001806276
PH_mr_0000422	hsa-miR-21-3p	141.7201153	86.87335882	1.631341498	-0.706058821	0.00026623
PH_mr_0002874	hsa-miR-21-5p	248.3130226	152.2078683	1.631407267	-0.706116984	0.002921658
PH_mr_0001414	hsa-miR-218-1-3p	121.1283037	79.69374239	1.519922394	-0.603997662	3.24813E-05
PH_mr_0000446	hsa-miR-22-3p	316.1448726	186.6700272	1.693602756	-0.760095523	0.006857838
PH_mr_0004651	hsa-miR-23c	132.029851	87.59132046	1.50733943	-0.592004326	5.04976E-05
PH_mr_0001658	hsa-miR-26a-1-3p	111.4380394	71.79616432	1.552144748	-0.634263105	6.93427E-05
PH_mr_0000750	hsa-miR-26a-5p	664.9943873	420.0075612	1.583291466	-0.662926864	0.007753401
PH_mr_0002298	hsa-miR-27a-3p	605.6415185	353.2371284	1.714546603	-0.777827118	0.000153456
PH_mr_0000433	hsa-miR-296-5p	140.5088323	90.46316704	1.553215932	-0.635258411	3.26071E-05
PH_mr_0000255	hsa-miR-299-3p	226.5099279	148.6180601	1.524107687	-0.607964841	1.67219E-06
PH_mr_0002338	hsa-miR-30a-3p	176.8473234	117.0277478	1.511157197	-0.595653744	0.001175481
PH_mr_0001970	hsa-miR-30b-3p	1895.657953	1176.739133	1.610941541	-0.687904142	3.23134E-05
PH_mr_0002420	hsa-miR-30c-1-3p	3567.228544	1881.777467	1.89566971	-0.922707619	1.45377E-05
PH_mr_0002352	hsa-miR-30e-3p	167.1570591	109.1301698	1.531721791	-0.615154282	0.041327237
PH_mr_0001890	hsa-miR-30e-5p	250.7355887	134.9767889	1.857620045	-0.893455445	0.001109493

PH_mr_0004600	hsa-miR-3127-3p	138.0862662	90.46316704	1.526436347	-0.61016743	0.000383657
PH_mr_0004128	hsa-miR-3129-5p	129.607285	82.56558896	1.569749415	-0.650534274	0.00090891
PH_mr_0004244	hsa-miR-3139	130.818568	81.12966568	1.612462801	-0.689265879	1.8566E-05
PH_mr_0004082	hsa-miR-3144-3p	134.4524171	82.56558896	1.628431636	-0.703483154	0.000805793
PH_mr_0004970	hsa-miR-3152-5p	138.0862662	90.46316704	1.526436347	-0.61016743	0.000108926
PH_mr_0004260	hsa-miR-3157-5p	152.6216627	99.7966684	1.52932623	-0.61289619	1.26924E-05
PH_mr_0004277	hsa-miR-3176	640.7687266	360.4167449	1.777855041	-0.830137697	0.004626974
PH_mr_0004040	hsa-miR-3178	2799.275098	1536.437916	1.821925291	-0.865463802	2.88599E-06
PH_mr_0004051	hsa-miR-3195	460.287554	264.2098847	1.742128439	-0.800850991	1.99243E-05
PH_mr_0004289	hsa-miR-3200-3p	133.2411341	87.59132046	1.521168232	-0.605179715	0.000151552
PH_mr_0002259	hsa-miR-320a	1396.609342	879.5030129	1.587952879	-0.667168103	5.82898E-05
PH_mr_0000512	hsa-miR-320c	1876.277424	940.5297525	1.994915545	-0.996327671	4.03795E-07
PH_mr_0000477	hsa-miR-324-5p	132.029851	84.00151225	1.571755645	-0.652376944	0.001975656
PH_mr_0000096	hsa-miR-329	125.9734358	80.41170403	1.566605724	-0.647642135	0.000256198
PH_mr_0003370	hsa-miR-331-3p	156.2555118	104.1044383	1.500949569	-0.585875504	0.000198365
PH_mr_0000216	hsa-miR-337-5p	136.8749832	90.46316704	1.513046554	-0.597456378	1.60658E-05
PH_mr_0002340	hsa-miR-33b-3p	132.029851	82.56558896	1.599090525	-0.677251613	1.16051E-06
PH_mr_0001107	hsa-miR-34b-5p	1078.041903	501.1372269	2.151191021	-1.105135639	0.000845231
PH_mr_0002170	hsa-miR-34c-5p	1124.070658	437.2386407	2.570840163	-1.362239916	1.25633E-05
PH_mr_0005109	hsa-miR-3607-3p	157.4667948	89.74520539	1.754598411	-0.811140867	3.36114E-07
PH_mr_0004976	hsa-miR-3618	135.6637001	76.82189582	1.765950953	-0.820445275	0.000418725
PH_mr_0004507	hsa-miR-3621	6098.810091	4059.35513	1.502408608	-0.587277234	5.75674E-05
PH_mr_0000129	hsa-miR-363-5p	4164.391081	1634.798661	2.547341871	-1.348992592	3.70659E-07
PH_mr_0004978	hsa-miR-3649	161.1006439	106.9762848	1.505947268	-0.590671254	0.001014625
PH_mr_0004982	hsa-miR-3658	117.4944546	73.2320876	1.604412197	-0.682044839	7.96229E-05
PH_mr_0002907	hsa-miR-367-3p	116.2831715	66.05247117	1.760466633	-0.815957884	0.000196328
PH_mr_0004986	hsa-miR-3671	145.3539644	89.74520539	1.619629303	-0.69566365	0.004499549
PH_mr_0004987	hsa-miR-3672	112.6493224	74.66801089	1.508669122	-0.593276432	2.15425E-06
PH_mr_0004669	hsa-miR-3679-5p	13699.61115	9075.753131	1.509473754	-0.594045672	0.000329359
PH_mr_0004514	hsa-miR-3687	406.9911004	224.7219943	1.81108708	-0.856855915	3.01606E-05
PH_mr_0004993	hsa-miR-3688-5p	117.4944546	73.2320876	1.604412197	-0.682044839	0.000311642
PH_mr_0002283	hsa-miR-369-5p	128.3960019	81.12966568	1.582602379	-0.662298831	6.03427E-05
PH_mr_0002318	hsa-miR-372	147.7765305	94.05297525	1.571205271	-0.651871675	0.00019178
PH_mr_0001189	hsa-miR-374a-5p	140.5088323	91.89909032	1.528946933	-0.612538334	6.90587E-05
PH_mr_0008164	hsa-miR-374c-3p	129.607285	76.82189582	1.687113857	-0.754557339	8.19316E-05
PH_mr_0000555	hsa-miR-376c-3p	159.8893609	99.7966684	1.602151289	-0.680010386	5.04569E-05
PH_mr_0000329	hsa-miR-377-3p	148.9878135	95.48889854	1.560263191	-0.641789409	9.82881E-05
PH_mr_0001978	hsa-miR-377-5p	136.8749832	85.43743554	1.602049293	-0.679918538	0.000609933
PH_mr_0000415	hsa-miR-381-3p	153.8329457	96.92482183	1.58713674	-0.666426429	8.49805E-05
PH_mr_0002851	hsa-miR-384	135.6637001	80.41170403	1.687113857	-0.754557339	3.59025E-05

PH_mr_0005005	hsa-miR-3942-5p	130.818568	73.2320876	1.786355849	-0.837019499	3.04589E-05
PH_mr_0005053	hsa-miR-3973	125.9734358	79.69374239	1.580719289	-0.660581191	0.000134276
PH_mr_0005054	hsa-miR-3974	127.1847189	84.00151225	1.514076538	-0.598438137	0.003198352
PH_mr_0000982	hsa-miR-409-5p	130.818568	81.84762732	1.598318391	-0.676554827	0.00012532
PH_mr_0000435	hsa-miR-410	122.3395867	73.95004924	1.654354365	-0.726268295	0.00075521
PH_mr_0003339	hsa-miR-424-5p	666.2056703	389.8531722	1.708863023	-0.77303676	0.000665702
PH_mr_0004317	hsa-miR-4260	148.9878135	95.48889854	1.560263191	-0.641789409	3.47282E-05
PH_mr_0004109	hsa-miR-4263	141.7201153	87.59132046	1.617969846	-0.694184721	0.002103576
PH_mr_0004107	hsa-miR-4266	140.5088323	83.28355061	1.687113857	-0.754557339	3.10905E-05
PH_mr_0004327	hsa-miR-4270	11491.44217	7640.547806	1.504007627	-0.588811883	8.89489E-06
PH_mr_0004143	hsa-miR-4272	119.9170207	72.51412596	1.653705662	-0.725702476	9.28661E-06
PH_mr_0004113	hsa-miR-4282	139.2975493	86.87335882	1.603455319	-0.681184153	0.000146451
PH_mr_0004336	hsa-miR-4286	8126.497895	3273.187131	2.482747722	-1.311937674	0.000299189
PH_mr_0004145	hsa-miR-4291	152.6216627	96.20686018	1.586390642	-0.665748072	2.61362E-05
PH_mr_0004133	hsa-miR-4303	136.8749832	83.28355061	1.643481602	-0.716755306	0.000454007
PH_mr_0000048	hsa-miR-431-5p	129.607285	81.12966568	1.59753259	-0.675845363	3.75633E-05
PH_mr_0004137	hsa-miR-4320	135.6637001	89.74520539	1.511654016	-0.596127976	0.000145782
PH_mr_0004057	hsa-miR-4321	185.3263047	113.4379396	1.633724178	-0.708164434	7.08935E-06
PH_mr_0004340	hsa-miR-4330	178.0586064	117.0277478	1.521507589	-0.60550153	5.68066E-05
PH_mr_0004704	hsa-miR-4417	324.6238539	190.2598354	1.706213259	-0.77079798	6.25896E-06
PH_mr_0004705	hsa-miR-4418	146.5652475	94.7709369	1.546521036	-0.629026457	0.000192167
PH_mr_0004718	hsa-miR-4432	150.1990966	96.20686018	1.561209838	-0.642664459	0.000237714
PH_mr_0004725	hsa-miR-4443	9926.464488	4302.744127	2.307007852	-1.206022914	0.000164129
PH_mr_0004730	hsa-miR-4448	2978.544988	1328.22904	2.2424935	-1.165103803	2.42518E-06
PH_mr_0004524	hsa-miR-4449	237.4114752	151.4899067	1.567176853	-0.648167994	0.000465406
PH_mr_0004733	hsa-miR-4454	13113.35016	5977.030679	2.193957311	-1.133535455	0.000122275
PH_mr_0005024	hsa-miR-4460	124.7621528	80.41170403	1.551542208	-0.633702944	0.000791273
PH_mr_0004528	hsa-miR-4466	787.333974	523.3940379	1.504285332	-0.589078243	6.25848E-05
PH_mr_0004740	hsa-miR-4472	2336.564978	1555.822881	1.501819396	-0.586711329	2.73176E-05
PH_mr_0005031	hsa-miR-4474-5p	129.607285	80.41170403	1.611796274	-0.688669403	6.51255E-05
PH_mr_0004748	hsa-miR-4484	6448.870889	3487.857662	1.848948986	-0.88670542	0.002887965
PH_mr_0004756	hsa-miR-4493	164.734493	106.2583232	1.550320842	-0.632566815	4.67868E-05
PH_mr_0004759	hsa-miR-4497	51691.5036	33039.87686	1.564518652	-0.645718858	0.000509159
PH_mr_0000485	hsa-miR-449a	654.09284	313.0312764	2.089544685	-1.063188611	0.000770707
PH_mr_0002253	hsa-miR-449b-5p	932.6879385	313.0312764	2.97953594	-1.57508765	0.003766231
PH_mr_0005043	hsa-miR-4501	156.2555118	102.668515	1.521941871	-0.605913258	0.000486723
PH_mr_0005225	hsa-miR-4509	125.9734358	70.36024103	1.790406542	-0.840287213	6.43037E-05
PH_mr_0004769	hsa-miR-4517	145.3539644	96.20686018	1.51084823	-0.595358744	0.003375176
PH_mr_0000212	hsa-miR-451a	1212.49432	104.8223999	11.56712994	-3.531959039	0.006059413
PH_mr_0001571	hsa-miR-455-5p	133.2411341	85.43743554	1.559517011	-0.641099289	0.001733801

PH_mr_0004548	hsa-miR-4634	994.4633734	463.8032215	2.144149345	-1.100405396	5.82276E-07
PH_mr_0004549	hsa-miR-4638-5p	2815.021778	1214.7911	2.317288773	-1.212437839	3.31277E-06
PH_mr_0005060	hsa-miR-4650-5p	136.8749832	89.02724375	1.537450531	-0.620539991	0.000148572
PH_mr_0004915	hsa-miR-4659a-3p	135.6637001	81.84762732	1.657515368	-0.729022247	0.000204573
PH_mr_0005065	hsa-miR-4662b	138.0862662	90.46316704	1.526436347	-0.61016743	0.000814901
PH_mr_0004556	hsa-miR-4663	162.311927	106.9762848	1.51727018	-0.601478009	0.000768791
PH_mr_0004558	hsa-miR-4665-5p	1104.69013	722.9873747	1.527952172	-0.611599385	1.6407E-05
PH_mr_0005066	hsa-miR-4666a-5p	140.5088323	86.87335882	1.617398408	-0.693675097	0.000243533
PH_mr_0004811	hsa-miR-4677-5p	127.1847189	77.53985746	1.640249583	-0.713915355	0.000178181
PH_mr_0005069	hsa-miR-4679	130.818568	86.87335882	1.505853691	-0.590581604	3.5748E-05
PH_mr_0005126	hsa-miR-4704-3p	127.1847189	78.97578075	1.610426864	-0.687443143	5.49606E-07
PH_mr_0004931	hsa-miR-4714-3p	136.8749832	86.15539718	1.588698882	-0.667845706	0.000154786
PH_mr_0004619	hsa-miR-4717-3p	210.7632484	137.8486355	1.528946933	-0.612538334	1.9102E-05
PH_mr_0004834	hsa-miR-4723-5p	4553.212936	2991.746167	1.521924883	-0.605897154	0.000106894
PH_mr_0004578	hsa-miR-4730	266.4822681	152.92583	1.742558913	-0.801207432	5.60448E-06
PH_mr_0004837	hsa-miR-4732-5p	4319.43531	2602.610956	1.659654625	-0.730883047	4.20392E-05
PH_mr_0005130	hsa-miR-4735-3p	111.4380394	73.2320876	1.521710538	-0.605693953	0.00190683
PH_mr_0004627	hsa-miR-4747-3p	146.5652475	89.74520539	1.633126214	-0.707636292	0.002641695
PH_mr_0004586	hsa-miR-4749-5p	11973.53282	7894.706228	1.516653372	-0.600891398	4.1758E-05
PH_mr_0005086	hsa-miR-4762-5p	140.5088323	91.89909032	1.528946933	-0.612538334	0.001414244
PH_mr_0004860	hsa-miR-4781-5p	116.2831715	68.92431774	1.687113857	-0.754557339	6.60652E-05
PH_mr_0004598	hsa-miR-4787-5p	27612.40811	18324.53502	1.506854503	-0.591540122	0.000654378
PH_mr_0004865	hsa-miR-4792	4172.870062	2479.121554	1.683205108	-0.751210988	9.7288E-07
PH_mr_0004867	hsa-miR-4797-5p	129.607285	82.56558896	1.569749415	-0.650534274	8.61308E-06
PH_mr_0005104	hsa-miR-4802-5p	138.0862662	81.12966568	1.702044068	-0.767268391	0.000120783
PH_mr_0000218	hsa-miR-487a	130.818568	84.71947389	1.544138106	-0.626801792	2.50269E-05
PH_mr_0002000	hsa-miR-488-3p	142.9313984	89.02724375	1.605479316	-0.683004078	3.51753E-05
PH_mr_0000497	hsa-miR-490-5p	148.9878135	99.07870675	1.503731916	-0.588547388	0.004700166
PH_mr_0002575	hsa-miR-491-3p	270.1161172	178.7724491	1.510949358	-0.595455307	0.01046796
PH_mr_0000226	hsa-miR-495-3p	136.8749832	85.43743554	1.602049293	-0.679918538	1.97423E-05
PH_mr_0000062	hsa-miR-498	288.2853628	178.0544875	1.619085072	-0.695178792	0.000817988
PH_mr_0000729	hsa-miR-499a-3p	130.818568	76.10393417	1.718946194	-0.781524387	0.000187421
PH_mr_0000079	hsa-miR-499a-5p	136.8749832	87.59132046	1.562654638	-0.643998964	0.00023024
PH_mr_0008052	hsa-miR-5003-5p	146.5652475	91.18112868	1.607407691	-0.68473589	6.65633E-06
PH_mr_0008146	hsa-miR-5011-3p	136.8749832	82.56558896	1.657772746	-0.72924625	0.003519347
PH_mr_0000781	hsa-miR-505-3p	107.8041903	67.48839446	1.597373758	-0.675701918	3.1278E-06
PH_mr_0003343	hsa-miR-507	128.3960019	76.10393417	1.687113857	-0.754557339	0.000238157
PH_mr_0004876	hsa-miR-5096	5594.916348	3388.060994	1.651362345	-0.723656714	0.00014941
PH_mr_0008021	hsa-miR-5100	4349.717386	1748.954563	2.487038531	-1.314428859	4.45665E-07
PH_mr_0000977	hsa-miR-513a-5p	5484.689591	3314.828906	1.654592061	-0.726475565	0.000160523

PH_mr_0000892	hsa-miR-513c-5p	261.637136	161.5413697	1.619629303	-0.69566365	0.000165903
PH_mr_0004290	hsa-miR-514b-5p	1090.154733	702.8844486	1.550972902	-0.633173481	4.35498E-05
PH_mr_0000404	hsa-miR-517a-3p, hsa-miR-517b-3p	150.1990966	96.92482183	1.549645321	-0.631938052	0.000368469
PH_mr_0000398	hsa-miR-517c-3p	151.4103796	93.33501361	1.622224863	-0.697973811	0.000149688
PH_mr_0001219	hsa-miR-518a-3p	152.6216627	99.7966684	1.52932623	-0.61289619	0.00010807
PH_mr_0002321	hsa-miR-518d-3p	115.0718885	76.10393417	1.512036004	-0.596492493	4.49318E-05
PH_mr_0001183	hsa-miR-518e-3p	138.0862662	77.53985746	1.780842405	-0.832559851	0.000183084
PH_mr_0001192	hsa-miR-519a-5p	144.1426814	89.74520539	1.606132392	-0.683590818	0.000376584
PH_mr_0008041	hsa-miR-5195-3p	914.5186929	528.4197694	1.730667068	-0.791328217	2.29753E-05
PH_mr_0000191	hsa-miR-519b-3p	128.3960019	79.69374239	1.611117737	-0.688061927	0.001338025
PH_mr_0000146	hsa-miR-519e-3p	140.5088323	91.89909032	1.528946933	-0.612538334	1.25307E-05
PH_mr_0000645	hsa-miR-520a-5p	132.029851	79.69374239	1.656715409	-0.728325797	0.005759903
PH_mr_0000177	hsa-miR-520f	136.8749832	84.71947389	1.615625982	-0.692093252	0.013787667
PH_mr_0000403	hsa-miR-521	134.4524171	84.71947389	1.587030832	-0.666330156	0.001134605
PH_mr_0002274	hsa-miR-526b-3p	132.029851	79.69374239	1.656715409	-0.728325797	0.000471865
PH_mr_0000378	hsa-miR-539-5p	127.1847189	79.69374239	1.595918513	-0.67438699	9.80048E-05
PH_mr_0002524	hsa-miR-548a-5p	139.2975493	86.15539718	1.616817446	-0.693156794	8.13255E-05
PH_mr_0002139	hsa-miR-548ah-3p	132.029851	81.12966568	1.627393013	-0.702562701	2.01372E-06
PH_mr_0008101	hsa-miR-548at-5p	124.7621528	80.41170403	1.551542208	-0.633702944	0.000592289
PH_mr_0000719	hsa-miR-548av-5p ,hsa-miR-548k	133.2411341	88.30928211	1.50880101	-0.593402547	0.000208574
PH_mr_0008706	hsa-miR-548az-3p	144.1426814	95.48889854	1.509522925	-0.594092667	6.87118E-06
PH_mr_0002447	hsa-miR-548b-5p	152.6216627	91.18112868	1.673829496	-0.743152576	0.00367454
PH_mr_0002136	hsa-miR-548d-3p	123.5508698	81.12966568	1.522881535	-0.606803719	0.007729325
PH_mr_0001981	hsa-miR-548h-5p	167.1570591	104.8223999	1.594669262	-0.673257237	0.005851088
PH_mr_0001982	hsa-miR-548l	151.4103796	94.05297525	1.609841467	-0.686918622	0.019658939
PH_mr_0000100	hsa-miR-556-3p	151.4103796	100.51463	1.506351658	-0.591058607	5.89452E-05
PH_mr_0008103	hsa-miR-5588-5p	132.029851	75.38597253	1.751384861	-0.808496146	0.001022241
PH_mr_0000630	hsa-miR-561-3p	141.7201153	90.46316704	1.566605724	-0.647642135	0.001255186
PH_mr_0002475	hsa-miR-563	127.1847189	71.79616432	1.77146955	-0.824946667	0.000984693
PH_mr_0002709	hsa-miR-567	124.7621528	81.84762732	1.524322169	-0.608167852	0.000758543
PH_mr_0008154	hsa-miR-5690	144.1426814	92.61705197	1.556329837	-0.638147847	0.000466911
PH_mr_0008128	hsa-miR-5692a	130.818568	86.87335882	1.505853691	-0.590581604	0.000239702
PH_mr_0002393	hsa-miR-586	135.6637001	86.87335882	1.561626049	-0.643049024	0.000108316
PH_mr_0001817	hsa-miR-590-5p	133.2411341	83.28355061	1.599849347	-0.677936057	3.3097E-05
PH_mr_0000370	hsa-miR-591	116.2831715	73.2320876	1.587871865	-0.667094498	2.49747E-05
PH_mr_0002694	hsa-miR-607	136.8749832	89.74520539	1.525150927	-0.608952017	0.001287041
PH_mr_0008677	hsa-miR-6073	153.8329457	94.7709369	1.623208029	-0.698847906	0.000241512
PH_mr_0008625	hsa-miR-6084	132.029851	86.87335882	1.51979678	-0.603878427	0.000645535

PH_mr_0008628	hsa-miR-6087	49937.56577	32568.89402	1.533290192	-0.616630769	1.48364E-06
PH_mr_0008629	hsa-miR-6088	27924.91913	17478.7762	1.597647273	-0.675948927	1.42485E-05
PH_mr_0008683	hsa-miR-6126	21918.16655	13263.6234	1.652502178	-0.724652174	1.17714E-05
PH_mr_0000583	hsa-miR-618	136.8749832	84.00151225	1.629434751	-0.704371582	0.008539536
PH_mr_0000620	hsa-miR-619	132.029851	82.56558896	1.599090525	-0.677251613	2.87763E-05
PH_mr_0001351	hsa-miR-623	162.311927	99.7966684	1.626426308	-0.701705457	3.16549E-05
PH_mr_0003127	hsa-miR-624-5p	141.7201153	91.89909032	1.54212751	-0.624922059	0.000362732
PH_mr_0002404	hsa-miR-625-5p	264.0597021	160.1054464	1.649286192	-0.721841764	0.001350428
PH_mr_0001785	hsa-miR-639	123.5508698	81.84762732	1.509522925	-0.594092667	0.000125809
PH_mr_0002273	hsa-miR-646	123.5508698	79.69374239	1.550320842	-0.632566815	0.000376841
PH_mr_0008692	hsa-miR-6505-5p	175.6360404	114.1559013	1.538562951	-0.621583474	3.56521E-05
PH_mr_0001046	hsa-miR-651	121.1283037	76.82189582	1.576741922	-0.656946542	5.6894E-05
PH_mr_0001125	hsa-miR-652-3p	138.0862662	81.84762732	1.687113857	-0.754557339	0.002654738
PH_mr_0001084	hsa-miR-660-5p	195.016569	116.3097862	1.676699574	-0.745624214	1.34928E-06
PH_mr_0001619	hsa-miR-663a	975.0828448	613.8572049	1.588452228	-0.667621702	9.64312E-06
PH_mr_0000829	hsa-miR-665	552.3450649	366.8783997	1.50552626	-0.590267872	3.88114E-05
PH_mr_0008651	hsa-miR-6724-5p	368.2300432	229.0297642	1.607782484	-0.685072239	7.95614E-05
PH_mr_0001714	hsa-miR-708-3p	139.2975493	79.69374239	1.747910753	-0.805631524	0.021992122
PH_mr_0002589	hsa-miR-759	147.7765305	96.92482183	1.524651041	-0.60847908	0.00024155
PH_mr_0008037	hsa-miR-873-3p	127.1847189	81.84762732	1.553920658	-0.635912843	2.13259E-05
PH_mr_0002610	hsa-miR-874	198.6504181	130.6690191	1.520256443	-0.604314703	0.000210045
PH_mr_0003139	hsa-miR-876-3p	134.4524171	85.43743554	1.573694438	-0.654155442	1.19626E-05
PH_mr_0003261	hsa-miR-876-5p	112.6493224	74.66801089	1.508669122	-0.593276432	6.32234E-05
PH_mr_0002427	hsa-miR-887	157.4667948	100.51463	1.566605724	-0.647642135	0.015844783
PH_mr_0003341	hsa-miR-888-3p	130.818568	81.84762732	1.598318391	-0.676554827	8.70984E-05
PH_mr_0000371	hsa-miR-889	127.1847189	84.71947389	1.501245381	-0.586159807	0.002590784
PH_mr_0002885	hsa-miR-891b	140.5088323	88.30928211	1.591099247	-0.670023829	0.000144766
PH_mr_0000045	hsa-miR-9-5p	132.029851	81.84762732	1.613117635	-0.68985165	0.000472349
PH_mr_0002041	hsa-miR-933	1608.583873	875.9132046	1.83646492	-0.876931338	2.30481E-06
PH_mr_0000621	hsa-miR-938	138.0862662	91.18112868	1.514417163	-0.598762666	8.2328E-05
PH_mr_0000757	hsa-miR-940	159.8893609	102.668515	1.557335868	-0.639080122	0.000448584
PH_mr_0001760	hsa-miR-944	133.2411341	86.15539718	1.546521036	-0.629026457	7.33544E-06
PH_mr_0000718	hsa-miR-96-5p	146.5652475	88.30928211	1.659681111	-0.730906071	2.33491E-05

Supplementary Table 6. Down-regulated miRNAs between human spermatogonia and round spermatids

		Normalized Intensity			log_2 (Ratio)	P-value (Differentially expressed)
**ID**	**Name**	**S2**	**S3**	**S2/S3**	**S3/S2**	**S3/S2**
PH_mr_0004114	hsa-miR-4288	1032.013147	1549.361226	0.666089438	0.58621219	0.000628096
PH_mr_0001186	hsa-miR-150-5p	525.696838	791.9116924	0.663832651	0.591108504	3.46251E-05
PH_mr_0005162	hsa-miR-3662	713.4457088	1090.583736	0.654187006	0.612224992	0.000828626
PH_mr_0004731	hsa-miR-4450	633.5010283	969.2482183	0.653600405	0.613519217	0.003539425
PH_mr_0004905	hsa-miR-4520a-3p	621.388198	951.2991772	0.653199554	0.61440429	3.42338E-05
PH_mr_0004212	hsa-miR-2278	1498.357117	2312.554453	0.647922956	0.626105821	7.24127E-05
PH_mr_0001807	hsa-miR-1224-3p	5777.820086	9040.573011	0.63909888	0.645888936	6.01068E-05
PH_mr_0004859	hsa-miR-4780	1790.276329	2827.332951	0.63320322	0.659259503	6.68187E-06
PH_mr_0008057	hsa-miR-5187-5p	641.9800096	1019.505533	0.629697426	0.667269326	3.59868E-05
PH_mr_0008654	hsa-miR-939-3p	2808.965363	4485.106385	0.626287343	0.675103371	0.002335158
PH_mr_0004592	hsa-miR-4763-5p	28385.20669	45475.69048	0.624184183	0.679956296	0.001412201
PH_mr_0003263	hsa-miR-1260a	26890.48342	43568.06639	0.617206262	0.696175395	2.30846E-05
PH_mr_0000117	hsa-miR-32-3p	1177.367112	1923.419242	0.612121937	0.708109023	0.000735582
PH_mr_0004235	hsa-miR-3127-5p	1699.430101	2781.383406	0.61100174	0.710751606	1.22572E-05
PH_mr_0004902	hsa-miR-4423-3p	672.2620855	1109.9687	0.605658597	0.723423304	1.39296E-05
PH_mr_0004925	hsa-miR-4695-3p	4333.970706	7268.643675	0.596255767	0.745996781	2.07251E-06
PH_mr_0005148	hsa-miR-3145-5p	3831.288246	6427.910591	0.596039443	0.746520291	0.000166506
PH_mr_0004085	hsa-miR-3149	2351.100375	3953.814769	0.594641002	0.749909151	0.000190558
PH_mr_0004887	hsa-miR-3622a-3p	15655.83325	26336.26899	0.594459043	0.750350681	6.37019E-06
PH_mr_0005266	hsa-miR-4764-5p	852.743258	1453.154366	0.586822211	0.769004617	5.03105E-05
PH_mr_0005201	hsa-miR-4456	4606.50939	8112.248606	0.567846181	0.816427912	6.155E-06
PH_mr_0004339	hsa-miR-4290	3477.593599	6142.161857	0.566183972	0.820657185	0.000429448
PH_mr_0004792	hsa-miR-4641	1551.65357	2744.767362	0.565313327	0.822877385	0.001007679
PH_mr_0001178	hsa-miR-1243	277.3838155	492.5216872	0.563191069	0.828303639	1.91351E-05
PH_mr_0004827	hsa-miR-4713-5p	11109.88802	19828.66466	0.560294312	0.835743249	6.24126E-08
PH_mr_0002504	hsa-miR-1238-3p	5632.466122	10098.13051	0.557773156	0.842249592	0.001250509
PH_mr_0008024	hsa-miR-3184-3p	10087.56513	18173.76307	0.555061992	0.849279188	6.05119E-06
PH_mr_0004638	hsa-miR-3120-5p	954.4910331	1734.59533	0.550267268	0.861795581	0.000133639
PH_mr_0004988	hsa-miR-3673	148.9878135	274.2613477	0.543232996	0.880356984	0.000116944
PH_mr_0008662	hsa-miR-6511a-3p	9508.57184	17641.7535	0.538981108	0.89169339	3.65835E-05
PH_mr_0001373	hsa-miR-135a-3p	446.9634406	863.7078567	0.517493777	0.950386582	5.72874E-05
PH_mr_0004201	hsa-miR-2116-3p	4828.174185	9398.117909	0.513738414	0.960894143	3.37425E-06
PH_mr_0008664	hsa-miR-6514-3p	1517.737645	2968.053433	0.511357925	0.967594635	1.20678E-05
PH_mr_0004820	hsa-miR-4701-5p	14938.75369	29491.71042	0.50654077	0.981249704	9.52088E-07
PH_mr_0004643	hsa-miR-3173-5p	2185.154599	4332.180555	0.504400629	0.987358019	1.67363E-05

PH_mr_0008618	hsa-miR-6072	3621.736281	7227.0019	0.501139522	0.996715774	6.39413E-06
PH_mr_0004155	hsa-miR-449b-3p	3639.905526	7330.388377	0.496550161	1.009988631	8.61602E-07
PH_mr_0008656	hsa-miR-3617-3p	1068.351639	2152.449006	0.496342369	1.010592483	4.17495E-05
PH_mr_0001774	hsa-miR-125b-1-3p	330.6802691	677.7557911	0.487904749	1.035328569	7.50177E-07
PH_mr_0008712	hsa-miR-6509-3p	3404.916617	7030.28041	0.484321594	1.045962765	0.00116849
PH_mr_0008716	hsa-miR-6130	2059.181163	4401.822834	0.467801918	1.096030319	0.000164294
PH_mr_0004287	hsa-miR-3198	942.3782028	2021.779987	0.466113132	1.101247935	2.94692E-07
PH_mr_0002598	hsa-miR-634	9227.554176	20441.8039	0.451406061	1.147502306	6.64367E-05
PH_mr_0008719	hsa-miR-98-3p	524.485555	1179.61098	0.444625867	1.169336211	3.13021E-05
PH_mr_0008717	hsa-miR-6508-5p	1640.077232	3730.528698	0.439636675	1.185616353	1.23262E-05
PH_mr_0003276	hsa-miR-664a-3p	1603.738741	3678.117498	0.436021618	1.197528431	0.013761991
PH_mr_0004084	hsa-miR-3146	580.2045747	1400.743166	0.414211962	1.271558878	2.3925E-06
PH_mr_0008715	hsa-miR-6128	800.6580874	2011.728524	0.397995096	1.32917744	0.020307332
PH_mr_0001363	hsa-miR-206	530.5419702	1522.796645	0.34839975	1.521184507	2.79743E-06
PH_mr_0000903	hsa-miR-1	237.4114752	777.5524595	0.305331778	1.711550344	0.000320368
PH_mr_0008048	hsa-miR-5194	1982.870332	6587.298076	0.301014211	1.732096497	7.46748E-08
PH_mr_0008641	hsa-miR-6514-5p	485.7244978	1825.776459	0.266037222	1.910299981	1.88169E-07
